# Pathophysiology of reactive oxygen species (ROS)

**DOI:** 10.1007/s00204-025-04276-w

**Published:** 2026-01-14

**Authors:** José Manuel Pérez de la Lastra, Celia María Curieses Andrés, Elena Bustamante Munguira, Celia Andrés Juan, Eduardo Pérez-Lebeña

**Affiliations:** 1 Institute of Natural Products and Agrobiology, CSIC-Spanish Research Council, Avda. Astrofísico Fco. Sánchez, 3, 38206 La Laguna, Spain; 2 Hospital Clínico Universitario de Valladolid, Avenida de Ramón y Cajal, 3, 47003 Valladolid, Spain; 3 Cinquima Institute and Department of Organic Chemistry, Faculty of Sciences, Valladolid University, Paseo de Belén, 7, 47011 Valladolid, Spain; 4 Sistemas de Biotecnología y Recursos Naturales, 47625 Valladolid, Spain

**Keywords:** Reactive oxygen species, Redox signaling, Oxidative stress, NADPH oxidases (NOX/DUOX), Mitochondrial ROS, Reverse electron transport, Endoplasmic reticulum, Peroxisomes, Peroxiredoxins, RNS/RSS crosstalk, Ferroptosis, Peroxynitrite, Redox biomarkers

## Abstract

Reactive oxygen species (ROS) are context-dependent mediators that function as second messengers at low, localized flux and as drivers of damage when production overruns buffering capacity. Outcomes are dictated by source identity, subcellular compartment and pulse kinetics—the “where–when–how much” rule. We synthesize advances (2015–2025) across principal generators—mitochondrial electron transport, NADPH oxidases, xanthine oxidoreductase and ER/peroxisomal oxidoreductases—to show how compartmental H_2_O_2_ microgradients encode reversible cysteine signaling, while iron-rich niches pivot chemistry toward peroxynitrite, Fenton-derived ^·^OH, lipid peroxidation and regulated cell death (apoptosis, ferroptosis, parthanatos). We integrate these mechanisms with endothelial dysfunction, innate immune priming, ECM remodeling and barrier failure across cardiovascular, metabolic, neurodegenerative, oncologic, pulmonary, renal and critical-illness contexts, emphasizing crosstalk with RNS/RSS and iron metabolism as key modulators. Methodologically, we advocate species-resolved, compartment-aware assessment—e.g., DHE → 2-OH-E⁺ HPLC for O_2·_−, targeted HyPer/roGFP-Orp for H_2_O_2_ and peroxiredoxin redox state—embedded in composite panels that pair flux with damage footprints and iron/ferroptosis metrics for attribution and trial guidance. Therapeutically, we argue against indiscriminate antioxidant loading in favor of node-specific, compartment-targeted modulation (NOX/NOS tuning, mitochondrial QC/RET tempering, ER redox control, iron/ferroptosis management, calibrated sulfur-axis support), implemented as time-staged sequences and titrated to biomarkers. Clarifying which species arise, where and when, reframes ROS from generic toxicity to precision redox modulation with translational impact.

## Introduction

Reactive oxygen species (ROS) are a heterogeneous group of molecules derived from the partial or complete reduction of oxygen, most notably superoxide (O_2·_−), hydrogen peroxide (H_2_O_2_) and the hydroxyl radical (^·^OH). Far from being mere toxic byproducts of aerobic metabolism, ROS act as ubiquitous redox signaling mediators that regulate processes as diverse as cell proliferation, differentiation, inflammatory responses, autophagy and programmed cell death. The delicate balance between their generation and removal—maintained by a complex network of enzymatic and non-enzymatic antioxidant systems—determines whether ROS fulfill physiological signaling roles or, conversely, trigger oxidative damage with pathological consequences (Sies 2017), Fig. 1.

**Fig. 1:**
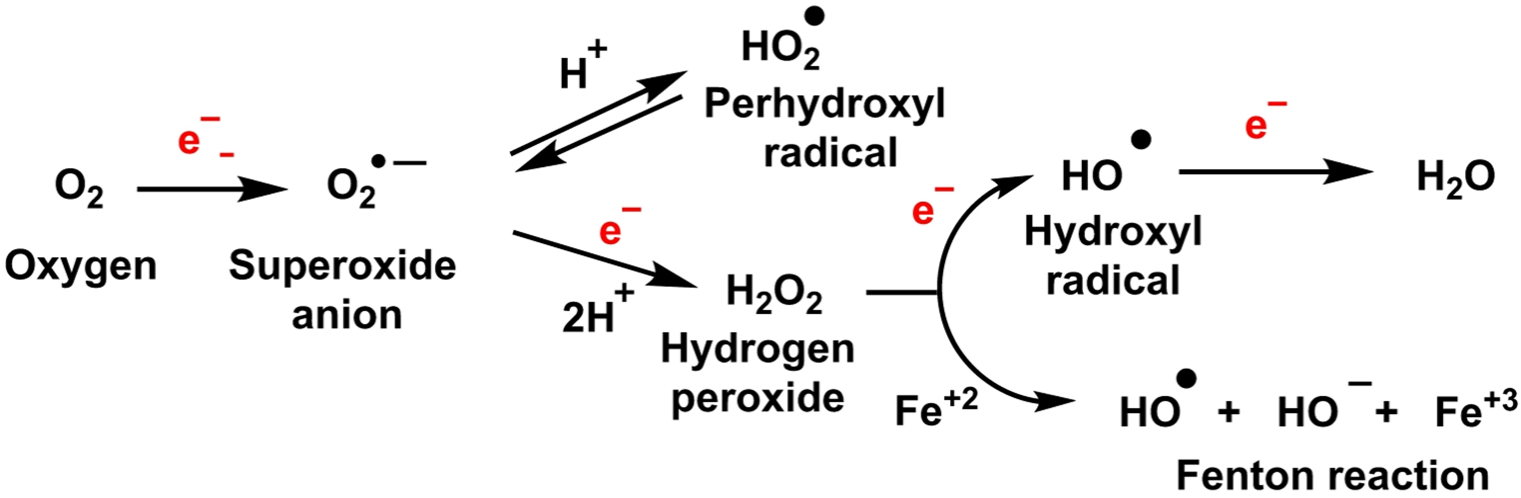
Formation of ROS, by sequential reduction of molecular oxygen

Under physiological conditions, the main intracellular sources of ROS include the mitochondrial electron transport chain, NADPH oxidases (NOX), xanthine oxidase and various endoplasmic reticulum and peroxisomal oxidoreductases. The subcellular localization of these sources and the chemical nature of the species produced confer specificity to redox signaling, particularly through reversible oxidation of sensitive cysteines on target proteins. Owing to its relative stability and diffusibility, H_2_O_2_ is recognized as a key second messenger capable of modulating pathways such as MAPK, PI3K/AKT and NF-κB. This physiological “redox tone” integrates with other layers of control, including mitochondrial bioenergetics, iron and glutathione metabolism and calcium homeostasis (Zhao et al. 2019), Fig. 2.

**Fig. 2:**
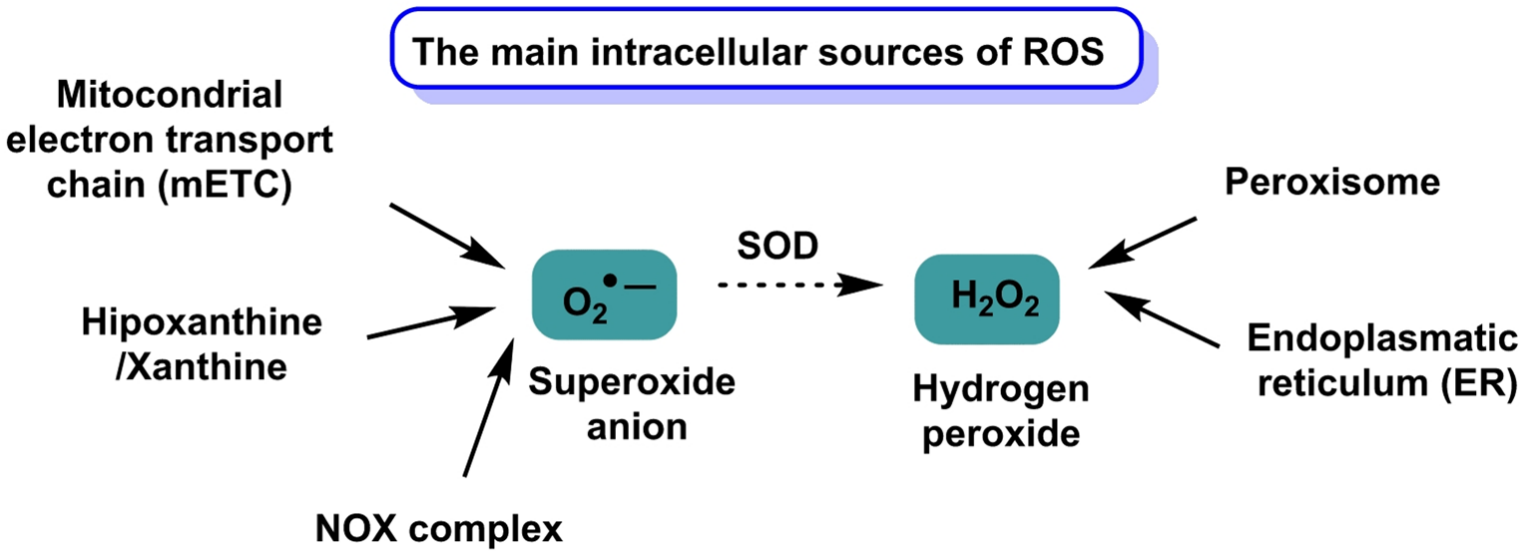
Main sources of ROS

When ROS production exceeds the buffering capacity of antioxidant systems, an oxidative stress state emerges. This favors irreversible modifications of macromolecules—lipid peroxidation, protein carbonylation and nitration, base oxidation and DNA strand breaks—that compromise the structural and functional integrity of cells and tissues (Juan et al. 2021). At the tissue level, ROS contribute to endothelial dysfunction, activation of innate immune cells, extracellular matrix remodeling and disruption of epithelial barriers. Systemically, these processes drive the progression of cardiovascular and metabolic diseases, neurodegenerative disorders, cancer and pulmonary and renal diseases, COVID-19 (Pérez de la Lastra et al. 2021), as well as phenomena related to aging. The intersection between redox signaling and cell death pathways—apoptosis, necroptosis, ferroptosis and parthanatos—represents a central axis of ROS-mediated pathogenesis (Marrocco et al. 2017), Fig. 3.

**Fig. 3:**
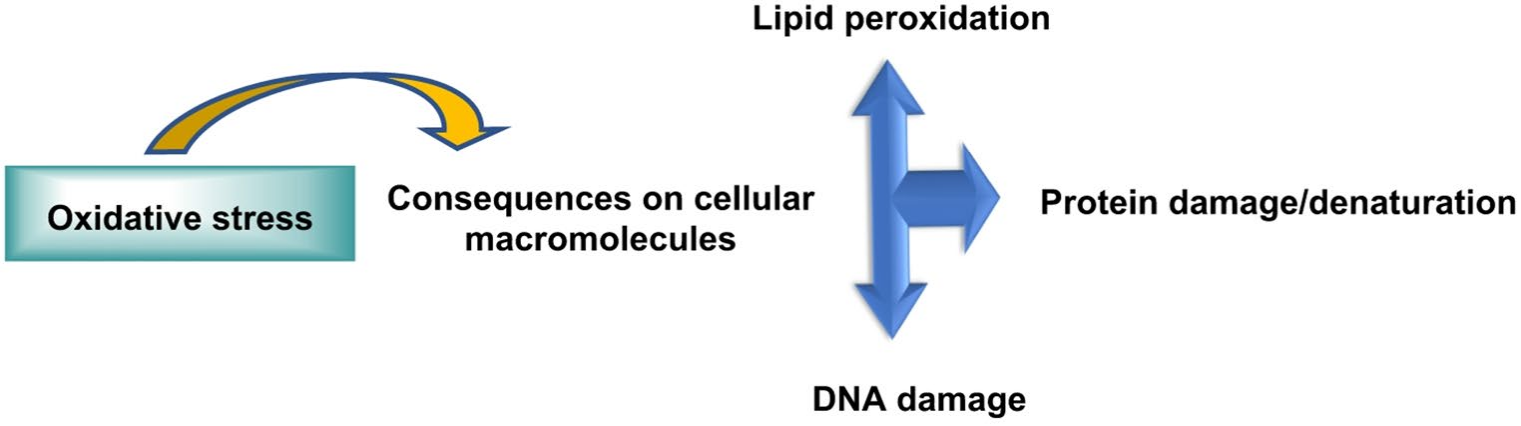
Consequences of oxidative stress on cellular macromolecules

The cellular antioxidant system comprises enzymes such as superoxide dismutases (SOD), catalase, glutathione peroxidases and peroxiredoxins, together with low-molecular-weight molecules (glutathione, uric acid, vitamins C and E, bilirubin and coenzyme Q). Their transcriptional regulation largely depends on the factor NRF2 and its repressor Keap1. Perturbations in this axis influence vulnerability to oxidative stress (Di Marzo et al. 2018), Fig. 4.

**Fig. 4:**
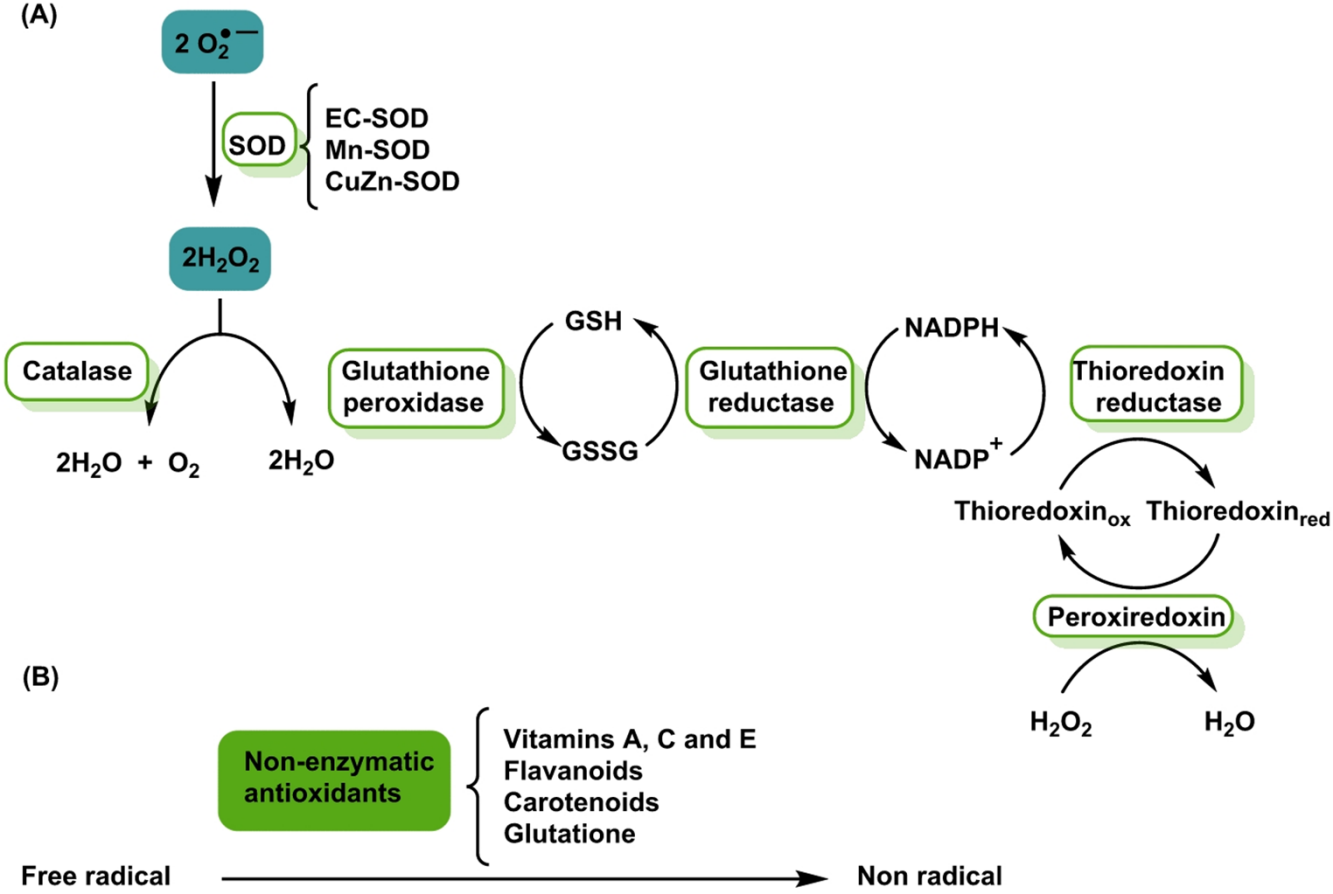
**A** The antioxidant enzyme system is composed of six enzymes (outlined in green in the figure). **B** Non-enzymatic antioxidants catalyse reactions to neutralise free radicals by donating electrons

However, therapeutic interventions based on nonspecific antioxidants have yielded mixed clinical results, underscoring that ROS biology is more a matter of “where, when and how much” than simple neutralization. Precise modulation of specific sources (e.g., NOX isoforms), restoration of mitochondrial biogenesis/mitophagy and subcellular targeting of redox agents are emerging as more rational strategies (Aisa-Álvarez et al. 2023).

The pathophysiology of ROS cannot be understood without considering their crosstalk with other reactive species—nitrogen (RNS), sulfur (RSS) and carbon—forming an interconnected redox network (Cortese-Krott et al. 2017). The formation of peroxynitrite (ONOO^−^) by the reaction of O_2·_− with NO^·^ exemplifies how vasoprotective nitric oxide signaling can be diverted toward cytotoxic routes. Similarly, the availability of labile iron conditions the propagation of Fenton-type reactions and susceptibility to ferroptosis, linking iron metabolism to oxidative vulnerability. These interactions, together with subcellular compartmentalization and microenvironmental gradients (hypoxia, pH and inflammation) determine highly contextual redox phenotypes (Prolo et al. 2024).

In this review, we first address the mechanistic principles of ROS generation, signaling and detoxification across key cellular compartments. We then integrate these principles with mechanisms of molecular damage and adaptive responses (e.g., mitochondrial and ER unfolded protein responses, autophagy and DNA repair). Next, we examine the evidence linking redox dysregulation to representative clinical entities—atherosclerosis, heart failure, type 2 diabetes, COPD, acute kidney injury, sepsis, neurodegenerative diseases and cancer—highlighting points of convergence and tissue specificity. Finally, we discuss precision redox therapeutics, functional biomarkers (e.g., oxidized-cysteine profiles, redox proteomics, GSH/GSSG ratio) and methodological challenges, including detection artifacts and in vivo experimental design (Tanabe et al. 2022).

Together, ROS act as dual modulators—physiological and pathological—whose impact depends on the spatiotemporal dynamics and architecture of cellular control networks. Clarifying these dependencies not only sharpens our understanding of redox biology but also opens opportunities for selective intervention in diseases driven by oxidative imbalance (Sies 2017). This Introduction sets the conceptual framework for the rest of the work and justifies the need for diagnostic and therapeutic strategies that account for source, compartment and context specificity in ROS signaling.

## Scope and structure of the review

This review synthesizes advances in ROS biology from roughly 2015–2025, integrating earlier seminal work to place new findings in context. It is a narrative, mechanism-focused synthesis—prioritizing studies with strong experimental design, species/compartment resolution and direct relevance to human pathophysiology—rather than a formal meta-analysis. The manuscript proceeds from fundamentals to translation and then to implementation.

“Biological sources and subcellular compartments of ROS” section maps biological sources and subcellular compartments of ROS (mitochondria, NOX/DUOX, xanthine oxidoreductase, ER, peroxisomes, cytosol/nucleus, and extracellular interfaces), highlighting dominant species, triggers, and local antioxidant networks. “Redox signalling versus oxidative damage: balancing physiological and pathological ROS” section formalizes the distinction between redox signaling and oxidative damage—the “where–when–how much” rule—linking species, source, compartment, and pulse design to molecular outcomes. “Methods for ROS Detection: Techniques, Limitations and Pitfalls” section reviews methods for ROS detection, with calibration, controls, compartment targeting, and common pitfalls.

“Crosstalk with RNS/RSS and Iron” section addresses crosstalk with RNS/RSS and iron (^·^NO/ONOO^−^ chemistry, persulfidation, Fenton/ferroptosis) and emphasizes microdomains and contact-site biology (MAMs, peroxisome–mitochondria, mitochondria–lysosome). “ROS in disease pathophysiology: cardiovascular, metabolic, neurodegenerative and oncologic disorders” section integrates these mechanisms across cardiovascular, metabolic, neurodegenerative, oncologic, and other disorders, linking mis-localization/mis-timing of flux to tissue phenotypes and motivating multi-parameter biomarker panels. “Therapeutic strategies targeting ROS: pharmacological and lifestyle interventions” section turns to therapeutic strategies that favor node-specific, compartment-aware interventions (NOX/NOS modulation, mitochondrial quality control and RET tempering, ER redox tuning, iron/ferroptosis control, and lifestyle levers) over indiscriminate scavenging.

Building on this, “Role of ROS in transcription regulation” section examines transcriptional regulation via localized, reversible cysteine chemistry on transcription factors, cofactors, and chromatin enzymes, including integration with RNS and RSS and guidance on how to measure transcription-linked redox with rigor and how to modulate nodes while preserving signaling.

“Biomarkers of ROS and clinical translation: from bench to bedside” section translates the framework into practice with clinically oriented biomarker panels—layering species-level reporters with damage footprints and iron metrics—and provides implementation pathways, validation standards, and disease-anchored examples (e.g., ICU use-cases and oncology/neuro/metabolic panels).

“Therapeutic perspectives” section surveys therapeutic perspectives and the translational landscape, aligning node- and compartment-targeted interventions with analytics that measure the right species in the right place/time and outlining safety, DDIs, and trial design considerations.

Recognizing that therapy is time-phased, “Combination and sequential therapies based on ROS tuning” section operationalizes combination and sequential strategies based on ROS tuning, defining acute/subacute/chronic windows, mechanism-guided combinations (source→sink/signal), disease archetypes, and biomarker-gated escalation/de-escalation.

“Knowledge gaps and future directions in redox biology” section delineates knowledge gaps and future directions—standards and calibrated, compartment-targeted in vivo sensors; contact-site mapping; iron–lipid redox homeodynamics and ferroptosis assays; human-proximal models; and mechanism-anchored trials with co-primary mechanistic and clinical endpoints.

Finally, Conclusions distills the central message: ROS are contextual signals whose impact depends on source identity, subcellular location, and pulse kinetics, demanding disciplined measurement and targeted modulation to achieve successful translation.

## Biological sources and subcellular compartments of ROS

Cells generate ROS at multiple enzymatic and non-enzymatic sites. Spatial segregation and kinetic control determine whether these species act as signals (e.g., H_2_O_2_ microgradients) or cause damage (e.g., ^·^OH near DNA). Below we map the principal generators, dominant species, triggers and compartment-specific antioxidant buffers, following the structure outlined in the manuscript plan and its updated table of contents (Hong et al. 2024).

### Mitochondria (matrix, inner membrane and intermembrane space)

Mitochondria are the most versatile intracellular sources of ROS. Electron “leak” from the respiratory chain reduces O_2_ to O_2·_−, which is rapidly dismutated to H_2_O_2_ (by SOD2 in the matrix and SOD1 in the intermembrane space). Production is highly site- and state-dependent (Hong et al. 2024), Fig. 5.

**Fig. 5:**
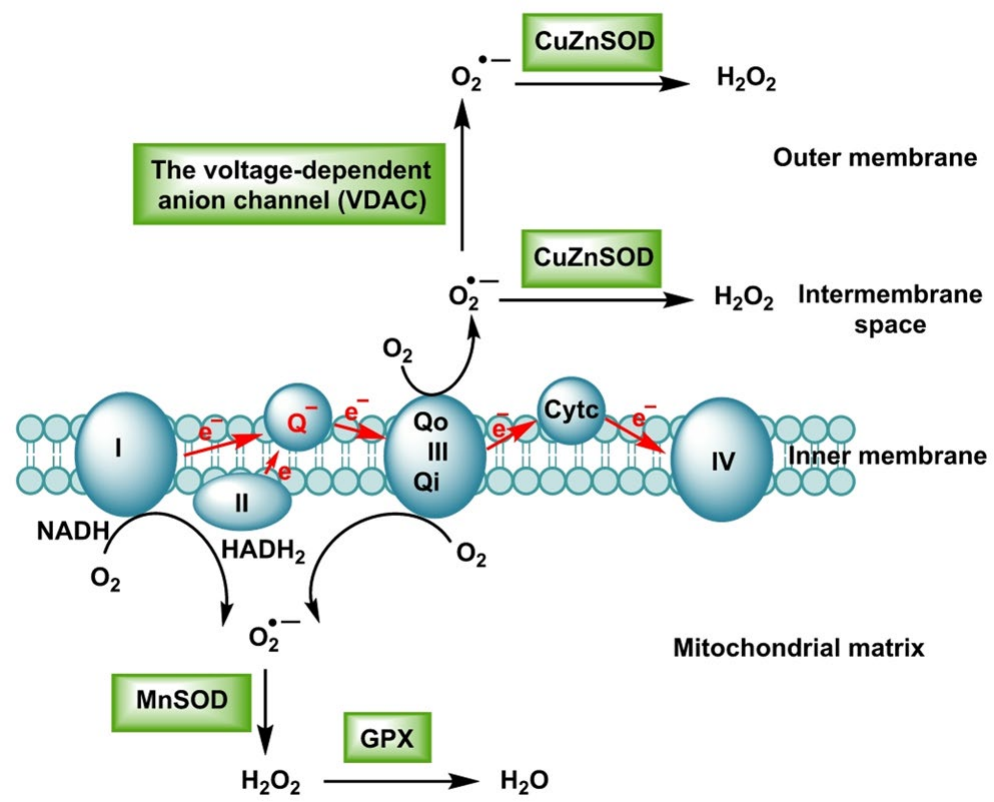
The superoxide anion is formed by monoelectronic reduction of oxygen, mainly in complexes I and III of the respiratory chain. The superoxide anion is dismutated to hydrogen peroxide by CuZnSOD in the intermembrane space and by MnSOD in the matrix

Mitochondrial ROS arise primarily as O_2·_− at the I_Q and III_Qo sites of the respiratory chain and are rapidly converted to H_2_O_2_ by SOD. Key triggers include a high membrane potential (ΔΨm) that favors electron leak, reverse electron transport (RET) during succinate overload, ischemia–reperfusion transitions with abrupt redox and calcium shifts, sustained Ca²⁺ overload, substrate re-routing that alters NADH/FADH_2_ supply and activation of the redox adaptor p66^Shc (Robb et al. 2018).

In the mitochondrial matrix, SOD2 together with Prx3, GPx, the GSH/GSSG couple and the Trx2/TrxR2 system confines H_2_O_2_ and supports controlled redox relays to metabolic enzymes and DNA repair machineries, whereas in the intermembrane space (IMS), SOD1, cytochrome c redox cycling and p66^Shc shape signals that can reach the cytosol. At the outer membrane/cytosol interface, H_2_O_2_ traverses peroxiporins (e.g., AQP8/3), enabling communication with cytosolic kinases and phosphatases without causing bulk oxidative stress (da Silva et al. 2024).

Amplex-type assays report pooled H_2_O_2_ and therefore integrate multiple mitochondrial and extra-mitochondrial sites, so accurate source attribution requires site-specific probes (including genetically encoded reporters targeted to matrix, IMS, or outer membrane) and carefully controlled substrate and inhibitor protocols that isolate individual electron-transfer nodes (Waldeck-Weiermair et al. 2021).

### NADPH oxidases (NOX/DUOX) at membranes and organelles

The NOX family comprises dedicated ROS-producing enzymes that channel electrons from NADPH to O_2_. Isoforms differ in subcellular targeting and regulation, shaping microdomain-level signaling (Cipriano et al. 2023b), Fig. 6.

**Fig. 6:**
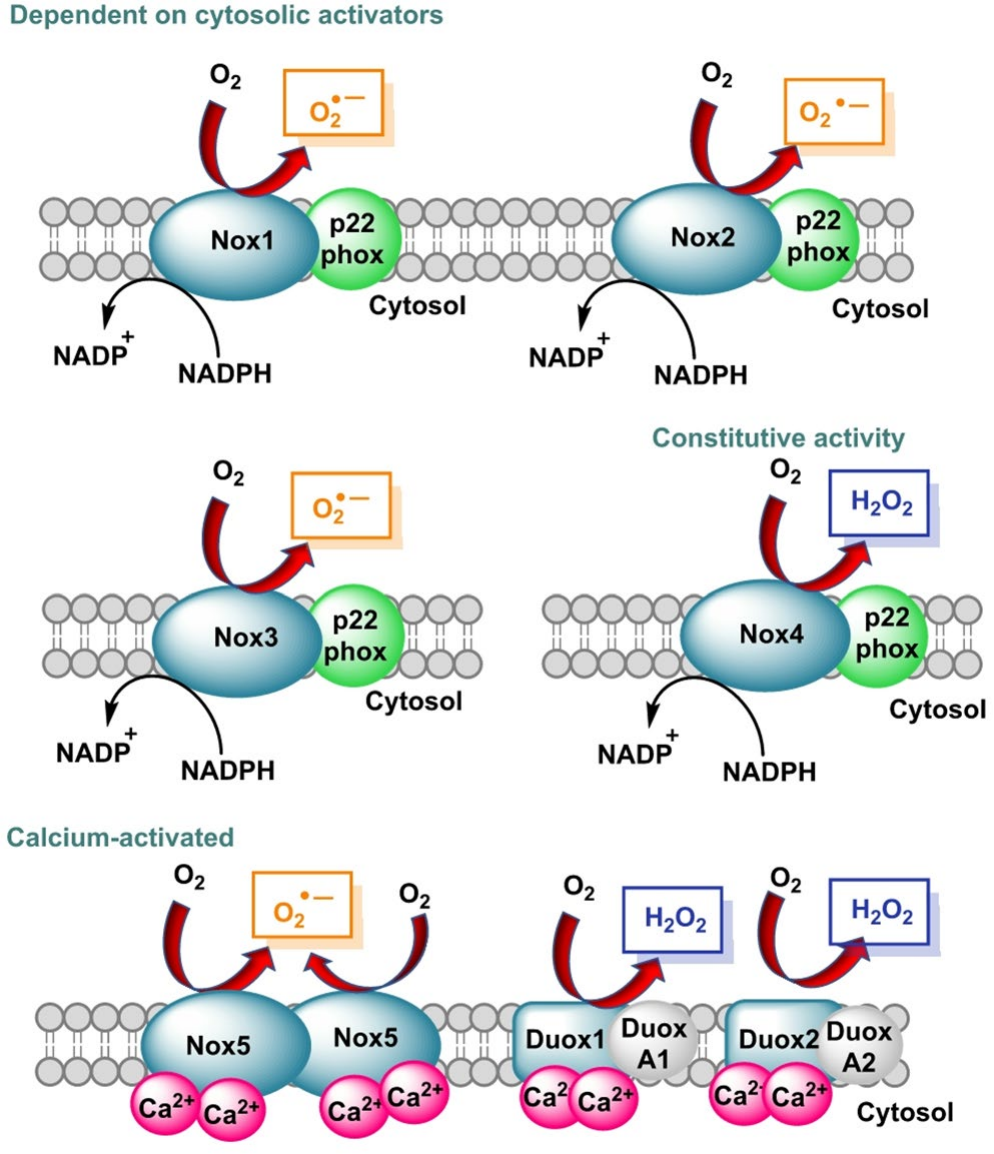
Classification of NADPH oxidase family members: Nox1-3 are activated by the assembly of cytosolic subunits and produce O_2·_−. Nox4 produces H_2_O_2_ directly. NOX5 and Duox can be activated by Ca²⁺ and produce O_2·_− or H_2_O_2_

NADPH oxidases generate ROS as O_2·_− or H_2_O_2_ depending on the isoform and context—NOX1/2/3 primarily release O_2·_− that dismutates to H_2_O_2_, NOX4 predominantly yields H_2_O_2_ and Ca²⁺-activated NOX5 together with epithelial DUOX1/2 contribute directly to H_2_O_2_ production at barrier surfaces (Kim and Moon 2024).

NOX complexes localize to the plasma membrane of endothelium and immune cells, but also to caveolae, focal adhesions, endosomes, the endoplasmic reticulum and nuclear envelope and even mitochondrial-associated membranes, creating microdomains where ROS can act with spatial precision (Cipriano et al. 2023b).

In health, tightly positioned NOX-derived H_2_O_2_ transiently oxidizes catalytic or regulatory cysteines on kinases and phosphatases to tune pathways controlling proliferation, migration and host defense, whereas chronic or dysregulated activity—driven by inflammatory cytokines, mechanical stress, hyperglycemia, or angiotensin II—amplifies redox tone, quenches nitric oxide and promotes endothelial dysfunction, vascular and tissue fibrosis and heightened innate immune priming (Kračun et al. 2025).

Attribution to specific NOX sources benefits from O_2·_− readouts by DHE-HPLC (especially for NOX2) and from compartment-targeted, genetically encoded H_2_O_2_ sensors such as HyPer variants that can be directed to the plasma membrane, endosomes, or nucleus to resolve isoform- and location-specific signaling with appropriate pharmacologic and genetic controls (Cipriano et al. 2023b).

### Endoplasmic reticulum (ER) and secretory pathway

Oxidative protein folding is a purposeful ROS source. Ero1 re-oxidizes PDI, passing electrons to O_2_ and producing H_2_O_2_. ER-localized peroxiredoxins (e.g., Prx4) and GPx7/8 detoxify and relay H_2_O_2_ (Zito et al. 2010).

In the endoplasmic reticulum (ER), the dominant ROS is H_2_O_2_, generated purposefully by oxidative protein-folding machinery and redox enzymes and. H_2_O_2_ output rises with high secretory load and activation of the unfolded protein response (UPR^ER) and is further modulated by Ca²⁺ fluxes as well as mono-oxygenase systems such as cytochrome P450 and cytochrome b₅ reductase (Roscoe and Sevier 2020). Crosstalk with mitochondria occurs at ER–mitochondria contact sites (MAMs), where tightly coupled Ca²⁺ and lipid exchange can propagate ROS signals bidirectionally—ER stress can potentiate mitochondrial ROS production and mitochondrial dysfunction can feed back to intensify ER oxidative pressure—shaping cell-wide redox tone and stress adaptation. Because bulk cytosolic readouts obscure compartmental signals, measurement should rely on ER-targeted genetically encoded H_2_O_2_ probes (e.g., roGFP-Orp1 or HyPer-ER), which report local peroxidatic events without cytosolic averaging and allow discrimination of ER-confined redox dynamics from extra-ER sources (Rashdan and Pattillo 2020; Zeeshan et al. 2016).

### Peroxisomes

Peroxisomes are H_2_O_2_ factories linked to fatty-acid oxidation and amino-acid catabolism. Acyl-CoA oxidases (β-oxidation) and D-amino acid oxidase produce H_2_O_2_ that is buffered by catalase and peroxiredoxins (He et al. 2021).

In peroxisomes, ROS output is dominated by hydrogen peroxide (H_2_O_2_) generated by flavin-dependent oxidases—most notably acyl-CoA oxidases during fatty-acid β-oxidation—while certain peroxisomal flavoproteins can also yield O_2·_− under specific redox conditions (Pascual-Ahuir et al. 2017).

H_2_O_2_ levels rise during surges of peroxisomal β-oxidation (e.g., fasting or PPARα activation), ether-lipid (plasmalogen) synthesis and polyamine detoxification via amine oxidases. Although peroxisomes are richly equipped with catalase (and peroxiredoxins such as Prx5) to shape the lifetime of H_2_O_2_, this oxidant can traverse peroxiporins to the cytosol, enabling peroxisome-to-cytosol redox signalling and metabolic coordination with mitochondria (e.g., matching fatty-acid flux with mitochondrial respiration) (Lismont et al. 2019). For measurement, organelle isolation often exaggerates apparent ROS production by disrupting matrix buffering and catalase activity, so in situ readouts with genetically encoded, peroxisome-targeted reporters (e.g., HyPer or roGFP-Orp1 fused to a PTS1/“SKL” signal) are preferred to capture authentic, compartment-confined dynamics (Fransen and Lismont 2024), Fig. 7.

**Fig. 7:**
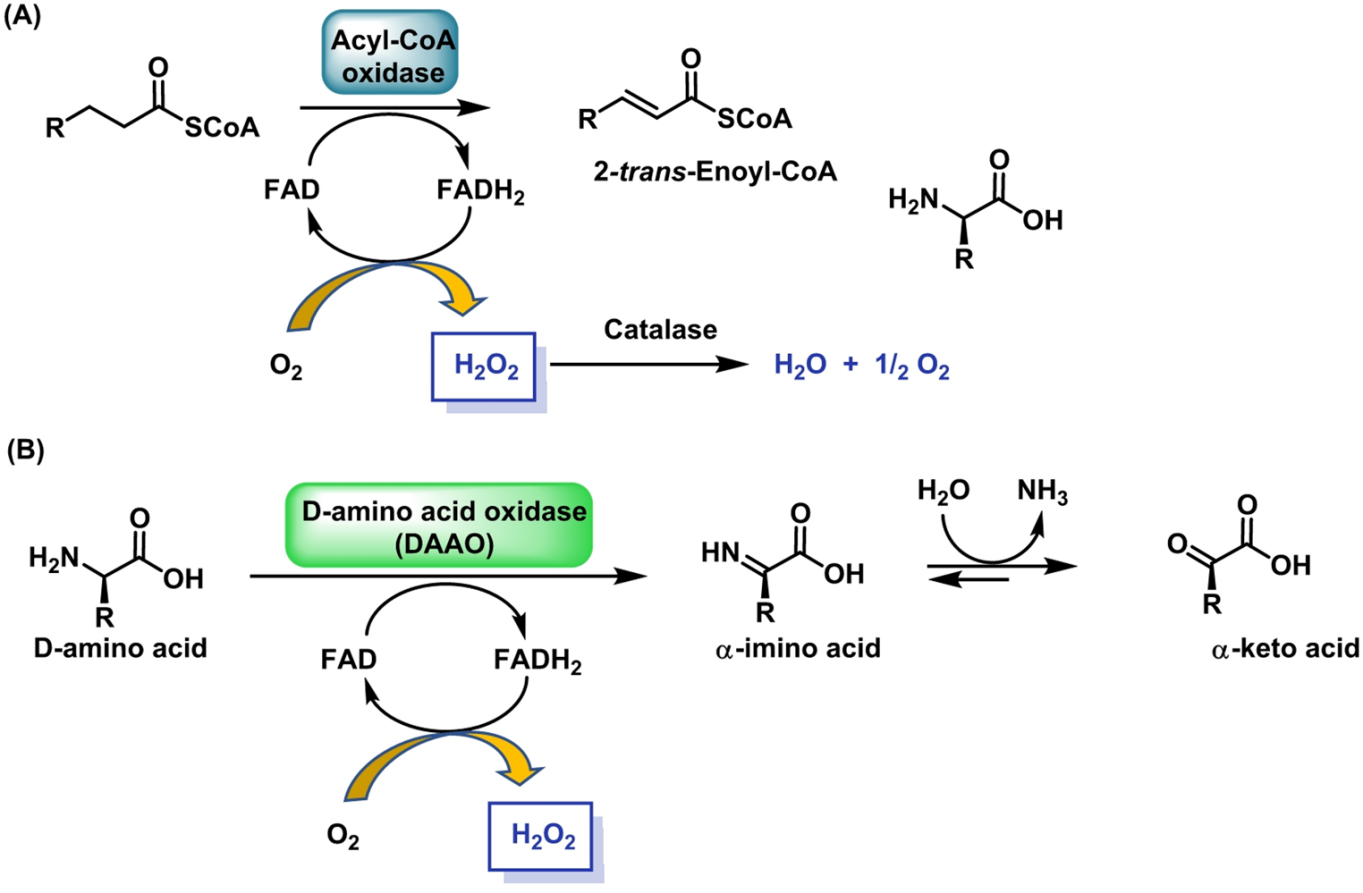
**A** FAD-containing acyl-CoA oxidases transfer protons from the β carbon bond of an activated fatty acid to molecular oxygen via a recycled FADH_2_ intermediate. **B** Amino acid oxidase is an enzyme that catalyses the oxidative deamination of amino acids, facilitating the removal of the α-amino group and converting it into ammonia

### Cytosol and nucleus

Although antioxidant systems keep basal ROS low, key cytosolic/nuclear enzymes generate localized signals. In the cytosol and nucleus, several enzymes create localized ROS microdomains: xanthine oxidoreductase (interconverting XDH and XO) at cytosolic and endothelial surfaces, monoamine oxidases on the outer mitochondrial membrane facing the cytosol, dihydrolipoamide dehydrogenase within the PDH/OGDH complexes and NOX isoforms positioned at the nuclear envelope (Moloney et al. 2017).

The dominant species are O_2·_− and its dismutation product H_2_O_2_, while nuclear ^·^OH arises secondarily through metal-catalyzed reactions in proximity to DNA or histone-bound metal centers. This chemistry sits directly at the genome interface: transient, targeted oxidation events modulate transcription factors and chromatin modifiers (e.g., redox-sensitive cysteines in DNA-binding proteins or histone-modifying enzymes), enabling physiological control of gene expression (Juan et al. 2021).

However, the same environment carries the risk of oxidative damage, such as 8-oxo-7,8-dihydroguanine (8-oxoG) and single- or double-strand breaks, which activate base excision and other DNA repair pathways. If not repaired or repaired incorrectly, they can alter transcriptional programs and genomic stability (Andrés et al. 2023a; Wang et al. 2025).

### Extracellular and membrane-proximal sources

At the organismal interface, ROS shape host defense and vascular tone. At organism-interface sites, extracellular and membrane-proximal ROS shape host defense and vascular tone: in professional phagocytes, NOX2 assembles at the phagosomal membrane to generate large bursts of O_2·_− into the lumen, where rapid dismutation and the action of myeloperoxidase convert upstream oxidants into hypochlorous acid (HOCl), a potent microbicidal species that collaborates with proteases and pH control to sterilize engulfed targets (Nauseef 2019).

Along the vascular lumen, endothelial XO/XDH and NOX1/NOX4 tune nitric-oxide bioavailability through competing O_2·_−/H_2_O_2_ fluxes—O_2·_− scavenges NO to form peroxynitrite (ONOO^−^), blunting vasodilation, whereas controlled H_2_O_2_ can serve as an endothelium-derived hyperpolarizing factor and redox cue for smooth-muscle relaxation—so that shifts in enzyme activity, shear stress, or metabolic inputs quickly translate into changes in vascular reactivity and oxidative burden (Konstandin et al. 2013).

At epithelial surfaces, secreted and membrane-anchored oxidases of the DUOX family deliver H_2_O_2_ into the mucosal milieu, where it supports barrier integrity, fuels lactoperoxidase-mediated antimicrobial chemistry and acts as a paracrine signal that coordinates wound closure and innate immune recruitment without triggering wholesale oxidative damage when spatially confined (Sarr et al. 2018).

### Non-enzymatic processes and metal-catalyzed chemistry

Non-enzymatic reactions become prominent when redox homeostasis is strained, creating ROS independently of dedicated enzymes: autoxidations arise as semiquinones and reduced flavins pass single electrons to O_2_, yielding O_2·_− that dismutates to H_2_O_2_ and seeds local chain reactions in metabolically intense niches (Hayyan et al. 2016).

**Fig. 8:**
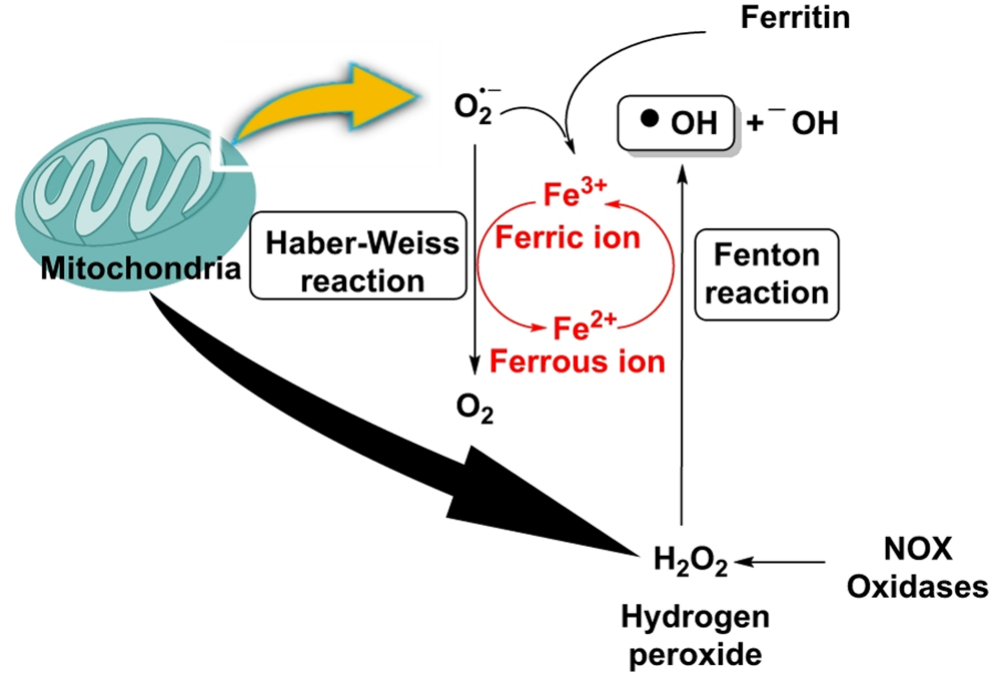
Fenton/Haber-Weiss reaction. Superoxide radicals produced in mitochondria can react with ferritin complexes, releasing ferric ions. These ferric ions can be reduced in the Haber-Weiss reaction to produce ferrous ions. Ferrous ions catalyse the Fenton reaction to generate ^·^OH from H_2_O_2_ produced by mitochondria and NOX oxidases

In Fenton/Haber–Weiss chemistry, labile Fe²⁺ or Cu⁺ catalyze the conversion of H_2_O_2_ into the hydroxyl radical (^·^OH), Fig. 8, an ultra-short–lived oxidant that inflicts site-specific macromolecular damage—DNA base modifications and strand breaks near metal centers, protein carbonylation and initiation of lipid peroxidation—with risk amplified by iron mobilization (e.g., ferritinophagy) and disruption of Fe–S clusters (Zhang et al. 2022).

Photosensitization/UV in exposed tissues generates singlet oxygen (¹O_2_) and secondary radicals via endogenous chromophores (porphyrins, flavins, melanin, lipofuscin), propagating oxidative injury to membranes and extracellular matrix (Fuentes-Lemus and López-Alarcón 2020).

These routes are highly contextual—governed by metal availability, pH, oxygen tension and chromophore burden—and often outpace classical antioxidant defenses at their point of origin, underscoring why metal chelation, control of the labile iron pool and photoprotection can be as critical as enzymatic antioxidant capacity in limiting collateral oxidative damage (Zhong et al. 2025).

### Compartment-tailored antioxidant networks

Compartment-tailored antioxidant networks sculpt ROS lifetime, diffusion radius and signaling specificity by matching oxidant production with locally optimized detox systems: in mitochondria. SOD2 converts O_2·_− to H_2_O_2_, which is then buffered and relayed by Prx3 and GPx in concert with the Trx2/TrxR2 couple and the GSH/GSSG pool, allowing controlled redox communication with metabolic enzymes and mtDNA repair without bulk oxidative stress (Gencheva and Arnér 2022).

In the ER/secretory pathway, Prx4 and GPx7/8 work alongside protein-disulfide isomerase (PDI) cycles to manage the purposeful H_2_O_2_ generated during oxidative folding, with only limited catalase present, so peroxidase-based redox relays carry much of the detox and signaling burden (Gansemer and Rutkowski 2022).

Peroxisomes rely on very high catalase activity, complemented by Prx5, to temper the substantial H_2_O_2_ flux from flavin oxidases during β-oxidation and other pathways, thereby controlling H_2_O_2_ leakage through peroxiporins to the cytosol (Lismont et al. 2019).

In the cytosol/nucleus, SOD1, Prx1/2, GPx1/4 and the Grx/Trx1 systems, supported by abundant GSH, confine ROS and tune thiol switches on enzymes and transcriptional regulators while DNA repair machineries such as OGG1 and the base-excision repair (BER) pathway continuously survey and correct oxidative lesions, together establishing a compartment-aware redox homeostasis that enables signaling yet limits collateral damage (Le Gal et al. 2021).

### Principles emerging from compartmentalization

Compartmentalization reveals unifying redox principles: microdomains and relays emerge as short-range H_2_O_2_ gradients are shaped and transmitted by peroxiredoxin-based cycles, allowing selective oxidation of target cysteines without elevating bulk oxidative stress (Sies 2017).

Vectorial flow replaces the old “free diffusion” view, as ROS are generated at membranes or enzyme clusters and then channeled along protein networks, redox relays and contact sites (e.g., MAMs) to reach defined effectors. The governing rule is “where, when, how much”, the identity of the source (e.g., NOX4 vs. complex III), its subcellular location (matrix, IMS, ER lumen, peroxisome) and the pulse’s timing and amplitude jointly decide whether ROS function as second messengers that tune metabolism and signaling or tip into cytotoxic chemistry that triggers lipid peroxidation, DNA damage and regulated cell death (Zhao et al. 2022).

## Redox signalling versus oxidative damage: balancing physiological and pathological ROS

Cells continuously generate ROS, yet most of the time these species act as context-limited signals rather than indiscriminate toxins. The balance hinges on *source identity*, *subcellular location*, *chemical species* and *time–amplitude* of the ROS pulse. Here we frame the mechanistic transition from physiological redox signalling to oxidative damage, establishing concepts and terminology used in later disease sections (Murphy et al. 2022a).

### What counts as “signalling”?

At physiological levels, ROS—especially H_2_O_2_—act as second messengers. They reversibly oxidize redox-sensitive cysteines on kinases, phosphatases, ion channels and transcriptional regulators, tuning pathway gain without exhausting antioxidant buffers. These reactions occur within microdomains created by localized sources (e.g., NOX4 at membranes, specific sites of the respiratory chain) and peroxiporin-gated H_2_O_2_ diffusion, ensuring target selectivity (Sies 2017).

### The cysteine “oxidation code”

Cysteine side chains sample a ladder of oxoforms with distinct kinetics and reversibility: thiolate (Cys–S^−^) → sulfenic (Cys–SOH) → disulfide (protein–S–S–protein or S–S–G) / sulfenyl-amide, with further over-oxidation to sulfinic (SO_2_H) or sulfonic (SO₃H) at higher oxidant loads. Signalling lives in the reversible zone (SOH/disulfide/mixed disulfide), resolved by thioredoxin/glutaredoxin systems and peroxiredoxin-mediated relays. Crossing into the irreversible zone marks the onset of damage and loss of regulatory control (Forman et al. 2017).

### Spatial and kinetic gating

Signalling stays separate from damage through three intertwined features: first, compartmentalization, whereby the mitochondrial matrix, intermembrane space (IMS), ER lumen, peroxisomes and plasma-membrane microdomains each host distinct chemistries and antioxidant buffers. Second, buffer proximity, as SODs, peroxiredoxins, GPx and local GSH pools are co-localized with ROS sources to sculpt H_2_O_2_ lifetime and limit its reach and third, pulse design, since short, low-amplitude bursts favor selective thiol switches, whereas sustained or repeated pulses saturate these buffers and allow oxidants to spread to vulnerable macromolecules (Winterbourn 2020).

### Tipping points: from tone to stress

The shift to oxidative stress occurs when ROS production exceeds antioxidant capacity or when detox enzymes are inactivated (e.g., peroxiredoxin hyperoxidation) or mislocalized. Additional triggers include RET at complex I, Ca²⁺ overload, unfolded-protein response (UPR^ER) and labile iron expansion that accelerates Fenton chemistry. Once thresholds are crossed, ROS escape their microdomains and initiate chain reactions (Harrison 2014).

### The damage landscape

The damage landscape is dictated by both the ROS species and their location: in lipids, H_2_O_2_-initiated or ^·^OH-driven peroxidation generates reactive aldehydes that crosslink proteins and disrupt membrane architecture (Liu et al. 2025). In proteins, irreversible modifications such as carbonylation, nitration and over-oxidation disable enzymes and chaperones, collapsing proteostasis (Korovila et al. 2017). In nucleic acids, 8-oxoG lesions, abasic sites and single- or double-strand breaks arise—often near metal centers bound to DNA—triggering repair programs like base-excision repair (BER) and PARP-dependent pathways that, when over-activated, consume NAD⁺/ATP and can precipitate bioenergetic failure and broader metabolic collapse (Banda et al. 2017).

### Cell-fate coupling

ROS act as both inputs and effectors of regulated cell death. Moderate, spatially confined ROS bias signal adaptation (mitohormesis, transcriptional reprogramming). Higher, persistent loads engage apoptosis (mitochondrial permeabilization), necroptosis (kinase-driven membrane rupture), ferroptosis (iron-dependent lipid peroxidation), or parthanatos (PARP over-activation and NAD⁺ collapse). Which pathway prevails depends on lipid composition, iron handling and the ROS source/compartment (Qi et al. 2025).

### Determinants of outcome: “where, when, how much”

Outcome is governed by three non-independent axes—where, when and how much: *where* the ROS originate and in which compartment (e.g., NOX microdomains versus mitochondria and ER versus peroxisome) sets the immediate molecular targets and the surrounding antioxidant context that shapes reactivity (To et al. 2020).

*When* exposure occurs as brief, pulsatile cues versus chronic, sustained elevations has opposite consequences for signalling circuits and cumulative damage accrual. *How much* oxidant is delivered follows a non-linear dose–response in which modest H_2_O_2_ enhances signalling through reversible thiol switches, whereas high local fluxes ignite injury chemistry, including ^·^OH generation from H_2_O_2_ at iron-rich sites (Goulev et al. 2017).

Practically, distinguishing signalling from damage relies on complementary readouts: signalling proxies include reversible cysteine oxidation captured by redox proteomics, the redox state of peroxiredoxins, compartment-targeted H_2_O_2_ reporters and phospho-signatures downstream of redox-sensitive kinases. Damage proxies include lipid-peroxidation adducts, protein carbonyls or nitrotyrosine, DNA oxidation and strand breaks and evidence of bioenergetic failure. Interpretation should remain rigorously compartment-aware and coupled to source attribution (e.g., NOX versus mitochondrial origin) to avoid conflating cause with consequence (Zhang et al. 2021), Fig. 9.

**Fig. 9:**
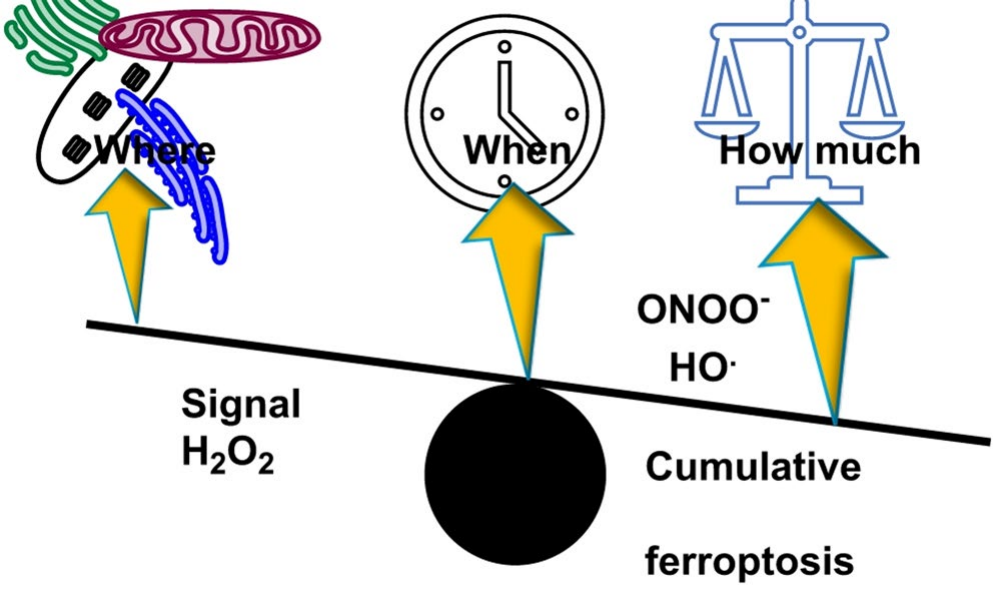
Practical readouts to distinguish redox signaling from damage

### Practical readouts to separate signalling from damage

Practical readouts to separate signalling from damage combine signalling proxies—such as reversible cysteine oxidation mapped by redox proteomics, the redox state of peroxiredoxins as fast cycling sentinels, compartment-targeted H_2_O_2_ reporters that resolve matrix/ER/PM microdomains and phospho-signatures downstream of redox-sensitive kinases—with damage proxies including lipid-peroxidation adducts, protein carbonyls or nitrotyrosine, DNA oxidation and strand breaks and indices of bioenergetic failure (Garrido Ruiz et al. 2022).

Critically, data must be interpreted in a compartment-aware manner and paired with source attribution (e.g., NOX versus mitochondrial origin) using targeted reporters, pharmacologic/genetic controls and site-specific sampling to avoid conflating adaptive signalling with downstream injury (Murphy et al. 2022a).

### Therapeutic implications

The biology argues against indiscriminate “antioxidant loading.” More promising are source- and compartment-targeted strategies (e.g., NOX isoform modulation, mitochondrial quality control, ER stress tuning), buffer re-balancing (GSH/thioredoxin systems), iron management to suppress Fenton chemistry and lifestyle interventions that reshape ROS tone without blunting adaptive signalling. These principles guide the disease-specific and therapeutic sections that follow (Meulmeester et al. 2022).

## Methods for ROS detection: techniques, limitations and pitfalls

Robust ROS quantification hinges on source attribution, compartment awareness and orthogonal validation. Throughout this section we emphasize how readouts should be matched to the dominant species and locale (“Biological sources and subcellular compartments of ROS–Redox signalling versus oxidative damage: balancing physiological and pathological ROS” sections) and interpreted against signalling-versus-damage criteria (“Biological sources and subcellular compartments of ROS” sections). We retain the manuscript’s structure and editorial conventions to ensure continuity.

### General principles and experimental design

Choose methods that (1) resolve the chemical species of interest (discriminating O_2·_− from H_2_O_2_ and from ^·^OH-derived footprints rather than treating “ROS” as a single entity), (2) report from the correct compartment (matrix vs. IMS, ER lumen, peroxisome, plasma-membrane microdomains, nucleus) and (3) embed specificity controls—SOD (for O_2·_−), catalase/GPx mimetics (for H_2_O_2_), metal chelators like DFO/TPEN (to suppress Fenton chemistry) and pathway inhibitors or genetic ablations (e.g., NOX isoform blockers, mitochondrial site inhibitors, siRNA/CRISPR) (Murphy et al. 2022a).

Wherever possible, calibrate with defined oxidant fluxes (enzymatic generators), internal standards, or ratiometric reporters and fully report matrix and environmental conditions (buffer composition, protein content, temperature, pH, oxygen tension, light exposure) plus acquisition timing/kinetics to separate bursts from steady states (Kalyanaraman et al. 2014, 2017).

Match sampling and normalization to the question (per mg protein, per cell, per tissue mass or surface, account for ΔΨm when using mito-probes), control for probe loading/photobleaching/pH sensitivity and verify that treatments do not alter probe uptake or peroxidase activity (Krishnamurthy et al. 2024).

Prefer live-cell, compartment-targeted readouts for signalling dynamics and pair them with biochemical footprints (lipid/protein/DNA oxidation) for damage attribution, include orthogonal genetic and pharmacologic controls and blinded analysis where feasible. Finally, combine at least two complementary assays (e.g., DHE-HPLC for O_2·_− and compartment-targeted HyPer for H_2_O_2_ and Prx redox Westerns and LC-MS damage markers) to avoid single-probe bias and to triangulate species, source and location with confidence (Stretton et al. 2020), Fig. 10.

**Fig. 10:**
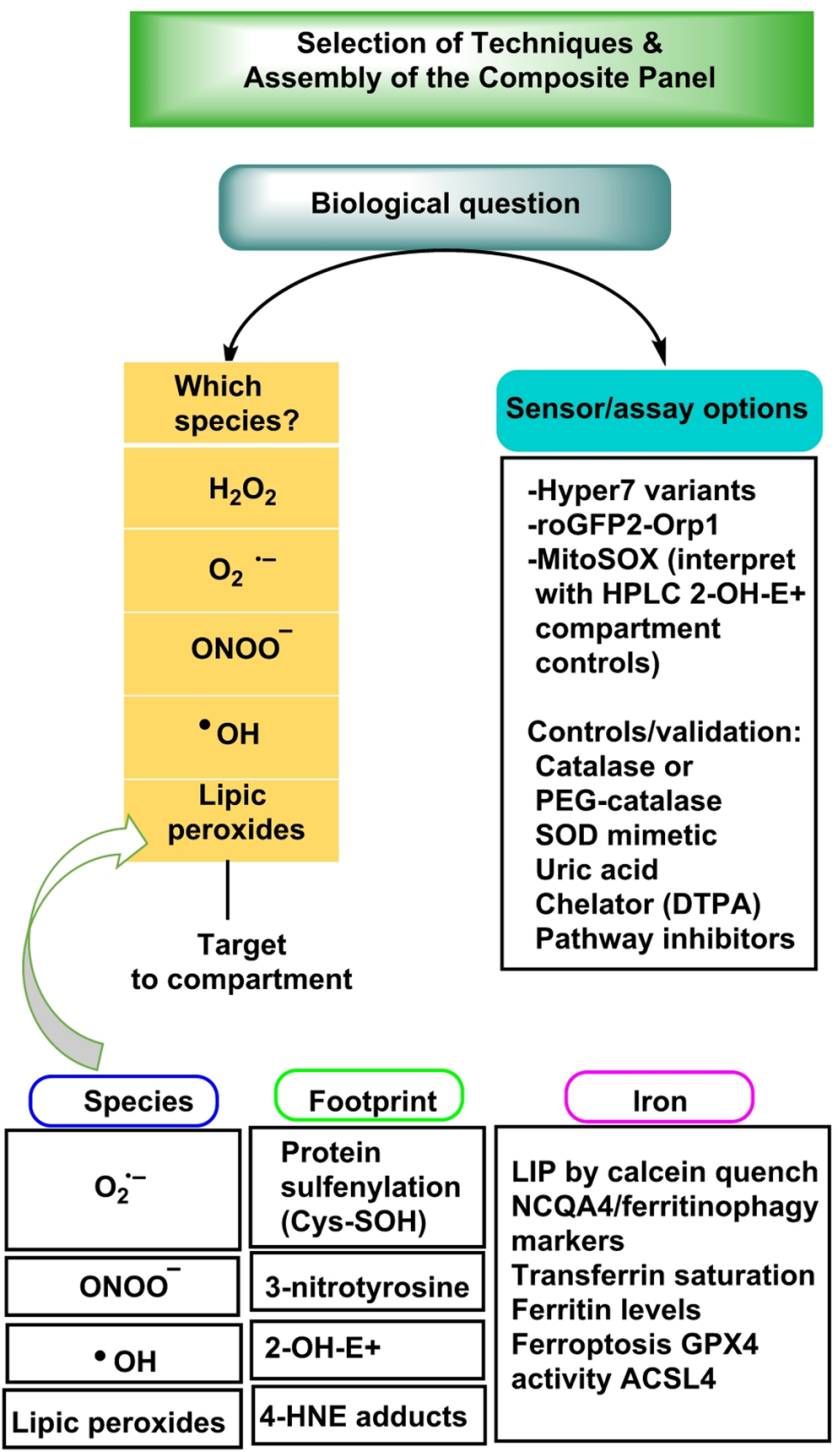
Orthogonal assays to triangulate ROS species, source, and compartment

### Small-molecule fluorescent/chemiluminescent probes

Small-molecule fluorescent/chemiluminescent probes are powerful but require disciplined controls: H_2_O_2_ reporters (Amplex-type, HRP-coupled) are highly sensitive for extracellular or compartment-averaged H_2_O_2_ yet pool signals from multiple sources and can be confounded by endogenous peroxidases and scavengers, so include catalase or catalase-mimetics as negative controls and state explicitly whether readouts reflect intra- versus extracellular H_2_O_2_ (Zhou et al. 1997).

Superoxide reporters of the DHE family yield non-specific raw fluorescence, thus quantification must rely on chromatographic separation of DHE→2-hydroxyethidium (2-OH-E⁺) by HPLC to measure O_2·_− and discriminate it from ethidium or other adducts, ideally alongside SOD controls and pathway-selective perturbations (e.g., NOX2 inhibitors, mitochondrial site inhibitors) for source assignment. Mitochondria-targeted probes (e.g., MitoSOX) can track mitochondrial O_2·_− trends but are susceptible to photobleaching, redox cycling and DNA intercalation, so interpret only with HPLC verification, strict light management and matched membrane-potential conditions (Zhao et al. 2005).

Across all probes, guard against general pitfalls—photo-oxidation, pH sensitivity, probe autoxidation and off-target enzyme catalysis—and always report probe concentration, loading time and washing protocol to support reproducibility (Zielonka and Kalyanaraman 2010).

### Genetically encoded redox reporters (compartment-targeted)

Target HyPer variants or roGFP-Orp1/roGFP2-Tsa2(ΔC) to the matrix, IMS, outer membrane, ER lumen, peroxisomes, nucleus, or PM microdomains to resolve localized H_2_O_2_ dynamics with minimal pooling (Malinouski et al. 2011). Calibrate dynamic range using oxidant/reductant pairs (e.g., diamide/DTT) and correct for pH shifts when applicable. Pair reporter data with source perturbations (NOX knockdown/isoform inhibitors, mitochondrial site blockers and Ero1 modulation) to make causal assignments (Nietzel et al. 2019).

### Protein-centric redox readouts

Protein-centric redox readouts provide fast, mechanistically anchored views of ROS signalling: the peroxiredoxin (Prx) redox state assessed by non-reducing SDS-PAGE or redox Westerns tracks Prx dimer formation and over-oxidation as a rapid sentinel of H_2_O_2_ flux and antioxidant buffer saturation (Stretton et al. 2020).

Redox proteomics of cysteines using isotope-coded or isobaric strategies (e.g., IAM/iodoTMT, OxICAT-like designs) quantifies reversible thiol oxidation (Cys-SOH and mixed disulfides), thereby mapping “signalling-zone” events (cf. “The cysteine “oxidation code”” sections) and enabling pathway-level analysis across protein networks and sulfenic acid capture with dimedone/DAz-2–type chemistries traps Cys-SOH in situ, but calls should be corroborated with orthogonal cysteine-state profiling (e.g., differential alkylation or redox-switch back-ups) to prevent over-interpretation (Anjo et al. 2019; Kisty et al. 2023; Vajrychova et al. 2019).

### Electron paramagnetic resonance (EPR) and spin trapping

EPR with spin traps (e.g., DMPO/DEPMPO families) provides direct radical evidence and kinetics but typically requires higher concentrations and controlled matrices. In cells or tissues, use membrane-permeant spin probes, strict oxygen and temperature control and metal chelation to reduce artifactual Fenton chemistry (Davies 2016).

### Footprints of oxidative damage (downstream proxies)

For downstream damage footprints, prioritize chemically specific assays: lipid peroxidation is best tracked by F_2_-isoprostanes and by MDA/HNE adducts—note that thiobarbituric acid–based colorimetry is prone to artifacts, so LC-MS/MS is preferred over simple spectrophotometric methods (Król and Brzeźnicki 2023).

Protein injury can be quantified via carbonyls (DNPH derivatization), nitrotyrosine as an RNS-linked marker and advanced oxidation protein products, with MS-based identification/validation wherever feasible to improve specificity and DNA damage should include 8-oxoG, abasic sites and strand breaks, interpreted cautiously because activation of repair pathways (BER/PARP) can secondarily reshape metabolic readouts (e.g., NAD⁺/ATP depletion) (Weber et al. 2015; Zhang et al. 2019a) .

Wherever possible, link these footprints back to species and compartment by pairing them with source-specific measurements (e.g., DHE-HPLC for O_2·_−, compartment-targeted H_2_O_2_ reporters, or iron-handling metrics) to avoid misattributing cause and consequence (Kalyanaraman et al. 2018).

### In vivo and point-of-care (POC) approaches

Bedside or minimally invasive POC strategies (electrochemical cartridges/biosensors and selected breath or surface readouts) can track trajectory and therapy response but rarely resolve species or compartments. Use them for trend monitoring and stratification, anchored to lab-grade or imaging-based assays for mechanistic depth. Always specify matrix, turnaround time, limit of detection and quality controls (Kim et al. 2024).

### Controls, calibration and reporting standards

Implement specificity controls (SOD, catalase/GPx mimetics, peroxiredoxin modulation, iron chelators), genetic tools (isoform knockdown/knockout, rescue) and pharmacologic orthogonality (distinct inhibitor classes). Provide time-stamped protocols, instrument settings and normalization schemes (per protein, per cell, per tissue mass, oxygenation). When feasible, share raw chromatograms/spectra (e.g., DHE-HPLC peaks) and calibration curves. Finally, align interpretations with the manuscript’s “where, when, how much” framework to avoid conflating adaptive signalling with downstream injury (Griendling et al. 2016).

## Crosstalk with RNS/RSS and iron

Redox networks do not operate in isolation. ROS are continuously shaped by—and shape in return—reactive nitrogen species (RNS), reactive sulfur species (RSS) and iron-centered chemistry (Cortese-Krott et al. 2017; Koskenkorva-Frank et al. 2013), Fig. 11.

**Fig. 11:**
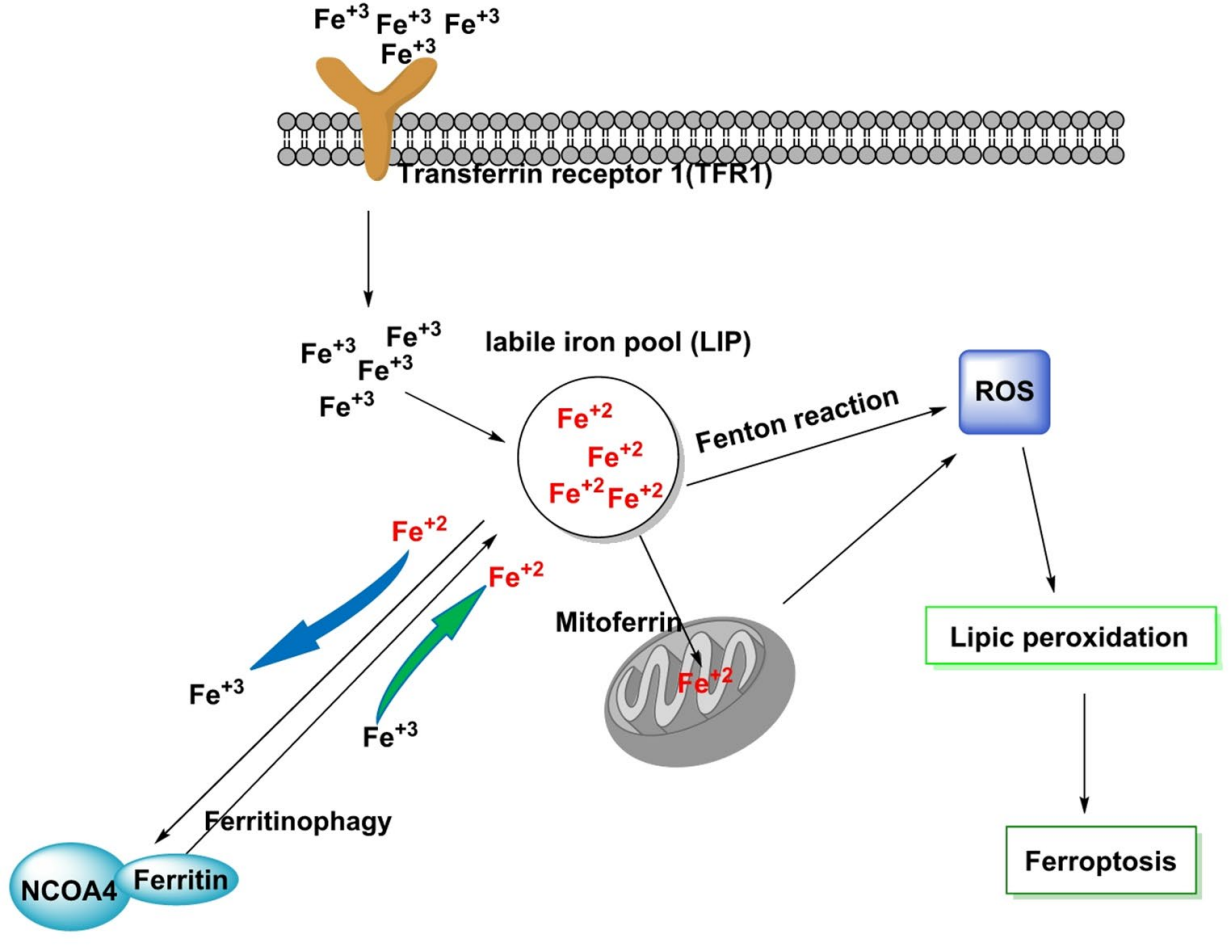
Iron metabolic pathway: Fe³⁺ is imported via transferrin receptor 1 (TFR1). Fe³⁺ is converted to Fe²⁺ and released into the cytoplasm. Fe²⁺ participates in the Fenton reaction, producing lipid ROS and causing ferroptosis. Ferritin stores iron and reduces Fe²⁺ to Fe³⁺, limiting the Fenton reaction. NCOA4 binds to ferritin, mediating its autophagic degradation in a process called ferritinophagy. This mechanism promotes ferroptosis. Iron from LIP can be transported to mitochondria via mitochondrial ferritin (mitoferrin) and stored in ferritin. Abnormal regulation of mitochondrial iron causes massive ROS production, which alters lipid peroxidation and promotes cellular ferroptosis

This section integrates the main mechanistic axes of that crosstalk and highlights why “mixed” redox biology often determines outcome more than any single species alone.

### ROS–RNS interface: NO biology, peroxynitrite and nitrosative stress

Nitric oxide (NO·) diffuses rapidly and competes with O_2_·− and their near diffusion-controlled reaction yields peroxynitrite (ONOO^−^/ONOOH) (Pérez de la Lastra et al. 2022), which nitrates and oxidizes targets (e.g., 3-nitrotyrosine, lipid peroxidation initiation) and can inactivate mitochondrial enzymes and peroxiredoxins. Beyond this chemistry, NOS–NOX reciprocity is pivotal: NOX-derived O_2_·− quenches NO bioavailability (Pacher et al. 2007), while NO can S-nitrosate NOX subunits and modulate activity. NOS uncoupling (e.g., tetrahydrobiopterin depletion) flips NOS from NO to O_2_·− production, amplifying ROS–RNS feed-forward loops (Kuzkaya et al. 2003).

GSNOR and thioredoxin systems tune the balance between reversible S-nitrosation (signalling) and irreversible nitration (damage). Methodologically, 3-nitrotyrosine is a useful but not fully specific footprint (requires LC-MS validation and protein-level mapping) and boronate-based probes detect ONOOH and H_2_O_2_ unless kinetic discrimination and scavenger controls are used (Benhar et al. 2009; Tsikas and Duncan 2014).

### ROS–RSS interface: H_2_S/polysulfides and the “sulfur buffer”

Endogenous H_2_S (CBS/CSE/3-MST pathways) (Andrés et al. 2023b)and reactive polysulfides (RSS) form a parallel signalling layer that intersects ROS at several levels. At physiological flux, H_2_S/RSS protect by (1) persulfidating catalytic cysteines (–SSH), stabilizing reversible thiol switches, (2) reducing sulfenylated intermediates (Cys–SOH) back to active thiols and (3) modulating mitochondrial electron flow via the SQR pathway (Andrés et al. 2025a).

Under oxidant excess (HOCl/ONOOH-rich niches), sulfur species are drained into higher oxidation products, attenuating persulfidation capacity and shifting the balance toward damage. The net effect is therefore context-dependent: in mild ROS fields, RSS preserve signalling fidelity, in harsh fields, they are consumed, losing cytoprotective potential. Detection must pair persulfidation chemistries (e.g., tag-switch) with orthogonal thiol-state profiling to avoid over-calling (Andrés et al. 2025a).

### Iron-centric redox: labile iron, Fenton chemistry and ferroptosis

The labile iron pool (LIP)—transient, chelatable Fe²⁺/Fe³⁺ not sequestered by proteins—acts as a catalytic hub that converts H_2_O_2_ into the hydroxyl radical (·OH) via Fenton/Haber–Weiss chemistry. Because ·OH has nanometer-scale diffusion and sub-microsecond lifetimes, it creates ultralocal damage microdomains at the sites where iron sits: genomic DNA (base oxidation/strand breaks near metal centers), Fe–S enzymes (cluster disruption with metabolic collapse) and PUFA-rich membranes (lipid peroxidation initiation) (Imlay 2003; Kakhlon and Cabantchik 2002).

The LIP is dynamically set by transferrin/TfR1 uptake (iron import), ferritin (safe storage via H-chain ferroxidase activity), NCOA4-mediated ferritinophagy (ferritin degradation releasing Fe²⁺), heme turnover by HO-1 (which can paradoxically raise LIP if not matched by storage/export) and mitochondrial–lysosomal exchange routes that shuttle iron for heme/Fe–S biogenesis. When iron availability and ROS converge on polyunsaturated phospholipids (PUFA-PLs), ferroptosis emerges: non-enzymatic and enzymatic lipid-peroxidation chains outpace detoxification by GPX4 (whose activity depends on selenocysteine and GSH), while ACSL4/LPCAT3 channel PUFAs into membranes and NOX/LOX activities (e.g., NOX1, ALOX15) help to seed peroxidation (Mancias et al. 2014; Stockwell et al. 2017).

Additional context setters include peroxisome-driven lipid remodeling and mitochondrial redox state and parallel anti-ferroptotic circuits (FSP1–CoQ10 at membranes, DHODH–CoQH_2_ in mitochondria) that can buffer propagation when intact. Thus, ROS sources (NOX complexes, mitochondria, peroxisomes) and iron handling co-operate to define ferroptotic susceptibility (Zou et al. 2020), Fig. 12.

**Fig. 12:**
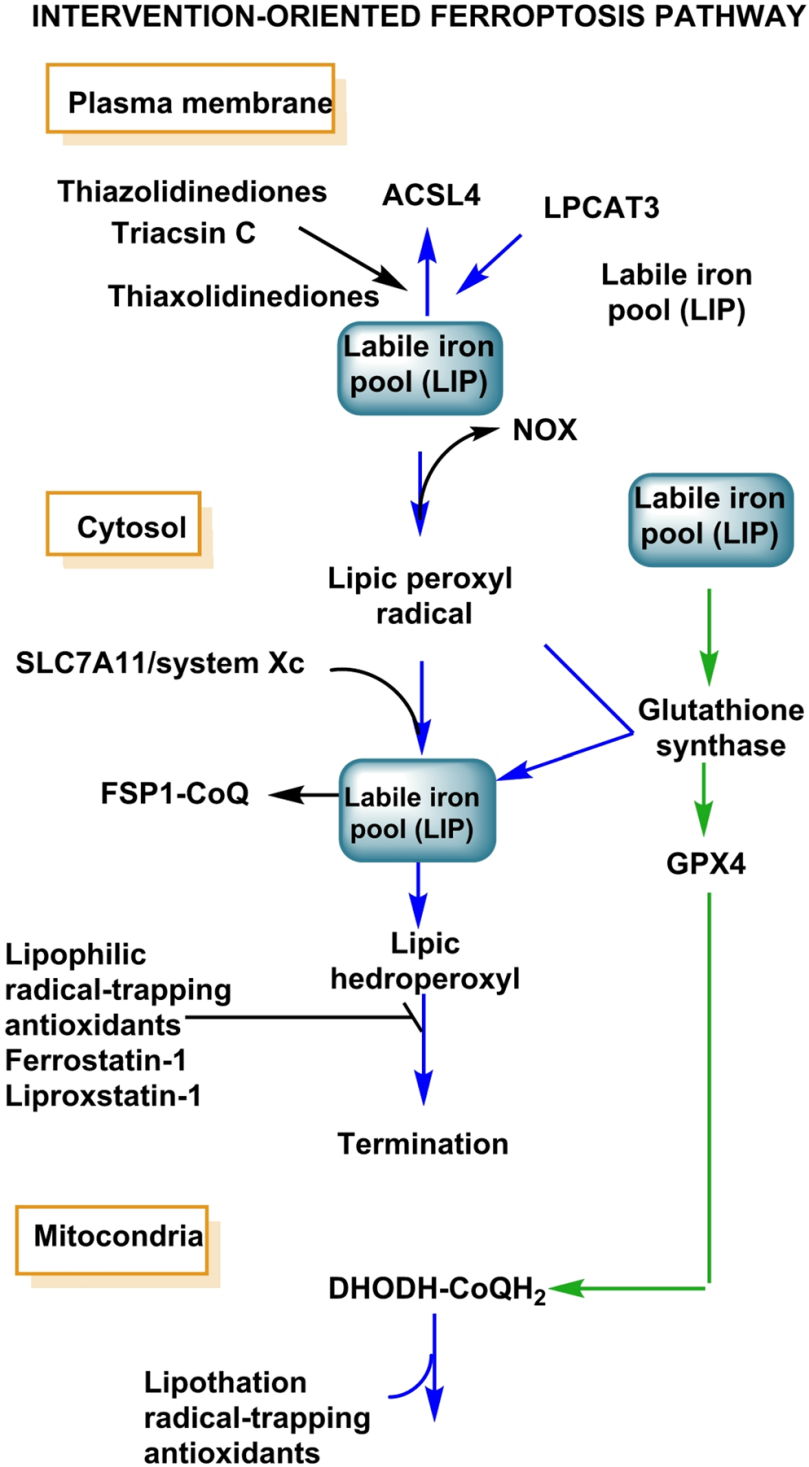
Integration of ROS sources with iron handling that sets ferroptotic susceptibility

Therapeutically, the axis can be interrupted by iron chelation (e.g., deferoxamine or deferiprone, with awareness of anemia/infection risks), radical-trapping antioxidants (ferrostatin-1, liproxstatin-1), selenium supplementation or GPX4 support (restoring the glutathione–GPX4 system), limiting LIP release by restraining ferritinophagy and, context-dependently, LOX/ACSL4 modulation or reinforcement of FSP1/CoQ10 defenses—always paired with biomarker guidance (LIP assays, ferritin/NCOA4/HO-1 status, lipid-peroxide readouts) to avoid blunting physiological iron/redox signalling (Mao et al. 2021; Zou et al. 2020).

### Spatial hubs for crosstalk: contacts and microdomains

Organelle contact sites are active hubs where multiple reactive chemistries are choreographed rather than isolated: at mitochondria–ER contacts (MAMs), tightly coupled Ca²⁺ and lipid transfer is synchronized with ROS generation and detoxification, so that ONOO^−^, H_2_O_2_ and RSS can be relayed across adjacent membranes via redox relays and peroxiporins, shaping metabolism, unfolded-protein responses and cell-death thresholds, at mitochondria–lysosome and peroxisome–mitochondria interfaces, fatty-acid flux, the labile iron pool (LIP) and ROS/RSS hand-offs are co-regulated—peroxisomal β-oxidation and catalase buffering, lysosomal iron mobilization and mitochondrial electron transport together set the likelihood of Fenton chemistry and lipid peroxidation (DiGiovanni et al. 2025; Yoboue et al. 2018), Fig. 13.

**Fig. 13:**
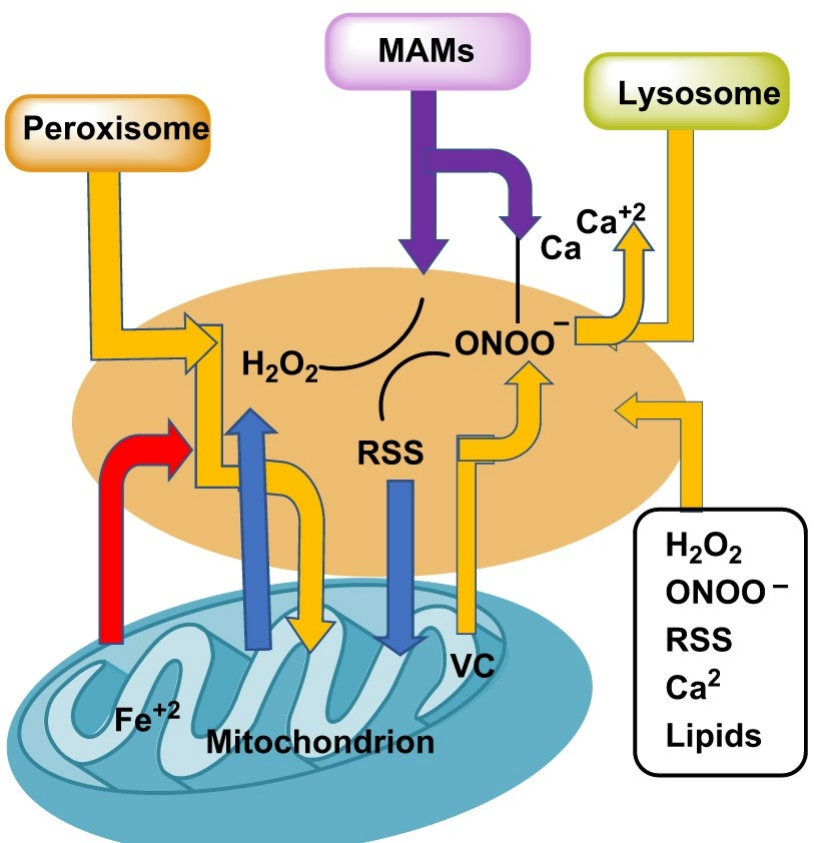
Redox at organelle contact sites and microdomains (MAMs, peroxisome–mitochondria, mitochondria–lysosome; plasma-membrane NOX/eNOS domains)

In the plasma-membrane plane, spatially organized NOX microdomains, eNOS signalling complexes and caveolae establish steep RNS/ROS gradients that tune nitric-oxide bioavailability, endothelial hyperpolarization and barrier function in a bed-specific manner. Mapping and perturbing these contact-site hubs—with compartment-targeted reporters, localized inhibitors/knockdowns and super-resolution/functional imaging—is essential for experimental design and for therapies that aim to modulate redox outcomes without blunting beneficial signalling (Leo et al. 2020; Yang and Rizzo 2007).

### Measurement and attribution across species

Because reactive species interconvert rapidly and share downstream products, multi-parameter panels provide far more reliable attribution than any single readout. In practice, combine species reporters that resolve flux and location—e.g., DHE-HPLC to quantify O_2_·− specifically via 2-OH-E⁺ (not raw fluorescence), compartment-targeted HyPer variants to track H_2_O_2_ in the matrix/IMS/ER/nucleus and NO/ONOO^−^ probes applied with kinetic discrimination and appropriate scavenger/enzyme controls—with footprints of damage that integrate exposure over time (3-nitrotyrosine for nitrative stress, lipid peroxides/F_2_-isoprostanes or MDA/HNE adducts for membrane injury and protein carbonyls for irreversible protein oxidation) (Murphy et al. 2022b; Zielonka et al. 2009) .

Layer these with iron metrics that determine ·OH propensity, such as calcein-quench assays for the labile iron pool (validated by reversible de-quenching with chelators) and markers of iron handling (ferritin levels, HO-1 induction and ferritinophagy readouts like NCOA4), because shifts in iron biology can transform benign H_2_O_2_ into damaging ·OH. To isolate sources and chemistries, apply genetic/pharmacologic separation: NOX and NOS isoform-selective inhibitors/knockdowns, modulation of CBS/CSE/3-MST for sulfur pathways and controlled iron chelation or loading to test Fenton dependence. Finally, enforce compartment targeting (organelle-addressed reporters, localized perturbations) and align temporal resolution (pulses vs. plateaus) so that species-level signals, cumulative footprints and iron status are read in context—avoiding the common pitfall of conflating correlated but mechanistically distinct redox events (Epsztejn et al. 1997; Mancias et al. 2014).

### Therapeutic implications: modulating nodes, not just scavenging

Therapy should target network nodes and compartments, not just mop up oxidants: indiscriminate “antioxidant loading” can blunt adaptive signalling and leave the upstream drivers untouched, whereas node-specific, compartment-aware strategies act where the chemistry originates and where signals are decoded. In the RNS axis, NOS re-coupling restores nitric-oxide production and curbs superoxide leak by supporting tetrahydrobiopterin (BH₄) availability, optimizing L-arginine flux, or limiting asymmetric dimethylarginine (Alp and Channon 2004; Meng et al. 2021).

Downstream, GSNOR/denitrosylation tuning rebalances reversible S-nitrosation without collapsing NO signalling. On the ROS source side, NOX isoform modulation (isoform-selective inhibitors or genetic silencing) dampens pathological microdomain fluxes while preserving host defense and organelle-targeted approaches (e.g., reinforcing mitochondrial quality control—biogenesis/mitophagy, precise control of Ca²⁺ handling and RET—rather than blanket scavenging) recalibrate matrix and IMS redox (Altenhöfer et al. 2012; Benhar et al. 2009).

In the sulfur network, RSS augmentation with well-behaved H_2_S/persulfide donors (controlled-release chemistries) can preserve thiol signalling by persulfidation and recycle sulfenylated switches, provided dosing respects tissue redox set-points. Because iron converts benign H_2_O_2_ into ·OH, iron management—judicious chelation (e.g., deferoxamine/deferiprone), restraining ferritinophagy (NCOA4) and correcting hepcidin–ferroportin imbalance—reduces Fenton hotspots and lowers the floor for oxidative injury (Dixon et al. 2012; Szczesny et al. 2014).

When ferroptosis is the execution pathway, pair GPX4 support (selenium, GSH restoration) with radical-trapping antioxidants (ferrostatin-1/liproxstatin-1), consider ACSL4/LOX restraint to limit PUFA-PL peroxidation and bolster parallel defenses (FSP1–CoQ₁₀ at membranes, DHODH–CoQH_2_ in mitochondria). Critically, interventions should be layered and time-stamped—aligned to the where, when and how much of each reactive family—to preserve physiological redox cues (e.g., H_2_O_2_-mediated signalling) while preventing convergence onto damaging chemistries (ONOO^−^ formation, Fenton ·OH, runaway lipid peroxidation) (Doll et al. 2019; Mao et al. 2021).

Biomarker guidance (Prx redox state, GSH/GSSG, LIP assays, cysteine-oxidation proteomics, compartment-targeted reporters) helps titrate dose, route and timing, minimizing off-target effects and avoiding the common pitfall of trading one pathology for another by over-suppressing beneficial stress responses (Murphy et al. 2022b).

## ROS in disease pathophysiology: cardiovascular, metabolic, neurodegenerative and oncologic disorders

ROS contribute to disease not merely by “more oxidants,” but by misplaced, mistimed and mis-sized fluxes that overwhelm local buffers and rewire signalling toward injury. Across systems, common convergence nodes include endothelium, innate immune priming and extracellular matrix (ECM) remodelling, with disease-specific modifiers (lipid composition, iron handling, mitochondrial quality control) dictating phenotype (Schieber and Chandel 2014). Below we integrate these principles with representative mechanisms and translational readouts, building on “Biological Sources and Subcellular Compartments of ROS–Crosstalk with RNS/RSS and iron” sections and the manuscript’s organizing framework.

Across cardiovascular, metabolic, neurodegenerative and oncologic disorders, ROS drive pathology less by sheer abundance than by misplaced, mistimed and mis-sized fluxes that overwhelm local buffers and redirect signalling toward injury (Curieses Andrés et al. 2023). In CVD, endothelial NOX/XO-derived O_2_·− diminishes NO and promotes ONOO^−^, while mitochondrial bursts during ischemia–reperfusion trigger cell death and fibrosis (Münzel et al. 2017).

In metabolic disease, nutrient overload and ER stress elevate H_2_O_2_/O_2_·−, coupling lipotoxicity to ferroptotic risk when labile iron expands (Schieber and Chandel 2014). In neurodegeneration, mitochondrial dysfunction, NOX activity and impaired mitophagy foster protein/lipid oxidation, ONOO^−^-driven nitration and iron-sensitized lipid peroxidation (Ischiropoulos and Beckman 2003). In cancer, the same H_2_O_2_ signals that sustain growth and EMT coexist with antioxidant rewiring, creating a therapeutic window where node- and compartment-specific interventions (NOX/NOS modulation, mitochondrial quality control, iron/ferroptosis management, calibrated RSS support) outperform blanket scavenging (Lankheet et al. 2015), Fig. 14.

**Fig. 14:**
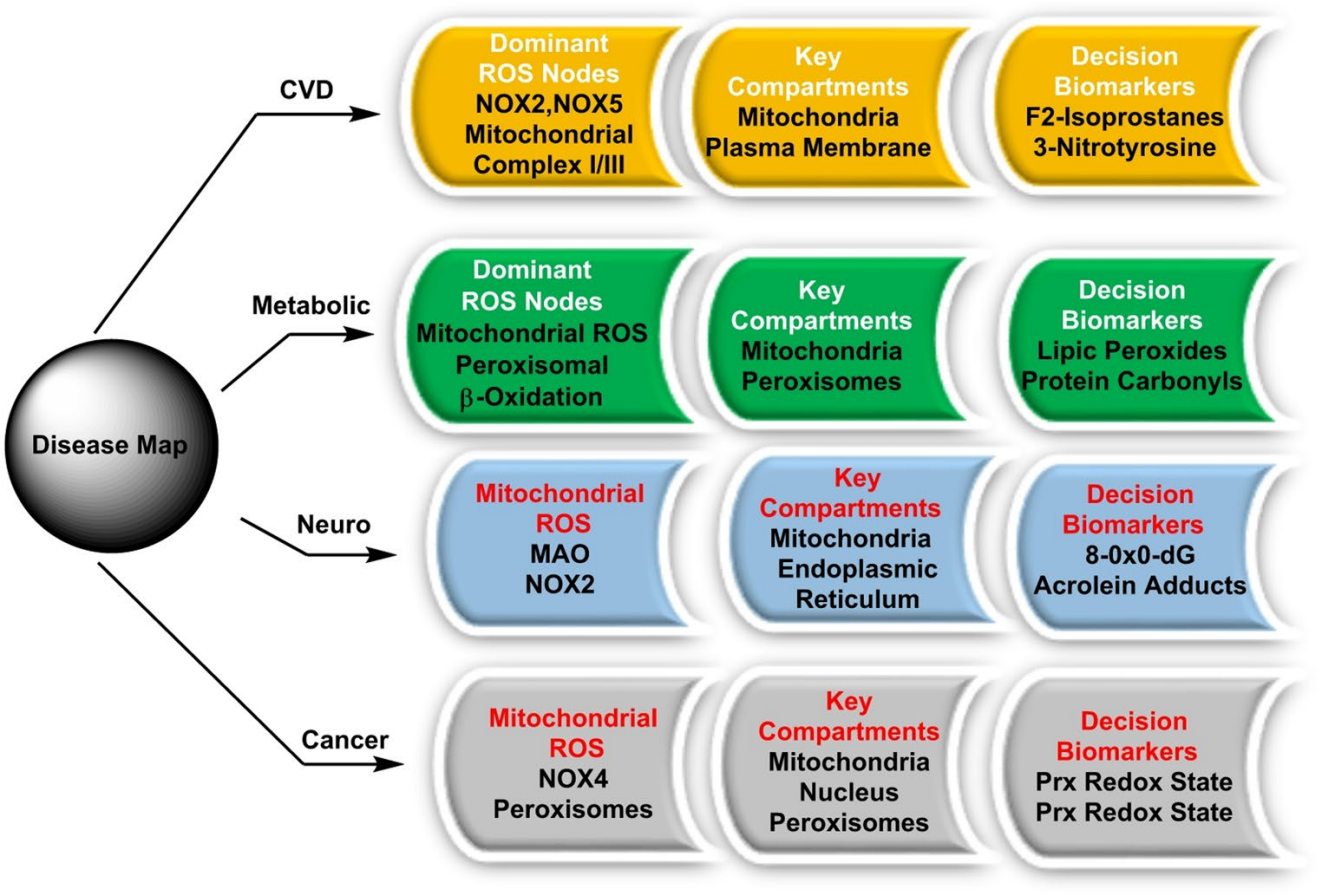
ROS-mediated signalling cascade

Clinically, multi-parameter panels that integrate species-level reporters with damage footprints and iron metrics—interpreted with strict source attribution and compartment awareness—enable mechanism-anchored stratification and monitoring, preserving beneficial H_2_O_2_ signalling while preventing convergence onto ONOO^−^, Fenton ·OH and runaway lipid peroxidation (Griendling et al. 2016).

### Cardiovascular disease (CVD): endothelium, myocardium and vessels

In the vasculature, NOX1/NOX2/NOX4 and xanthine oxidoreductase generate ROS at the luminal face, where O_2_·− quenches NO and favors ONOO^−^ formation, impairing vasodilation and promoting leukocyte adhesion. Within the myocardium, mitochondrial ROS spikes—particularly during ischemia–reperfusion and reverse electron transport—trigger mPTP opening, Ca²⁺ mishandling and cell death programs (Drummond and Sobey 2014; Radi et al. 1997).

Chronic low-grade H_2_O_2_ biases hypertrophic signalling and fibrosis via redox-sensitive kinases and TGF-β crosstalk (Liu and Desai 2015). In atherogenesis, endothelial dysfunction and oxidized lipids drive monocyte recruitment and foam-cell formation and ferroptotic susceptibility rises in PUFA-rich plaques when labile iron expands (Ouyang et al. 2021). Clinically, pairing flow-mediated dilation or vascular reactivity tests with NOX/mitochondrial source markers and lipid peroxidation products strengthens mechanism-to-biomarker links and can guide node-specific therapy (e.g., NOX modulation, iron management, mitochondrial conditioning) (Griendling et al. 2016) .

### Metabolic disease: insulin resistance, NAFLD/NASH and complications

In adipose and liver, nutrient excess and ER stress amplify H_2_O_2_ production (Ero1–PDI axis, NOX4), while mitochondrial substrate overload raises matrix ROS and activates JNK/IKK pathways that blunt insulin signalling. Hepatic lipotoxicity couples peroxisomal β-oxidation (H_2_O_2_) with mitochondrial ROS and—when labile iron is available—drives lipid peroxidation and cell-death switching toward ferroptosis, accelerating NASH (Yu and Song 2024).

In pancreatic β-cells, limited antioxidant capacity makes protracted H_2_O_2_/O_2_·− exposure particularly injurious, undermining insulin secretion. Translationally, redox proteomics (cysteine-switches in insulin signalling), Prx redox state, F_2_-isoprostanes/HNE adducts and LIP assays (with chelator validation) help stratify patients and monitor interventions such as mitochondrial quality control, NOX isoform targeting and dietary/exercise prescriptions that reset redox tone without erasing adaptive signalling (Holendová et al. 2024).

### Neurodegeneration: redox–proteostasis–mitochondria triad

Neurons operate close to an oxidative edge because they consume large amounts of O_2_, are enriched in polyunsaturated lipids that are easy substrates for peroxidation and rely on long axons studded with far-flung mitochondria that are hard to replace. In this setting, mitochondrial ROS from complex I/III sites, NOX2/NOX4 activity in microglia and cerebrovascular endothelium and impaired mitophagy (reduced removal of damaged mitochondria) converge to oxidize proteins and lipids, seed misfolding and aggregation (e.g., α-synuclein, Aβ) and blunt synaptic plasticity by altering redox-sensitive receptors, kinases and Ca²⁺ handling (Lee et al. 2021).

At higher flux or with nitric-oxide present, ONOO^−^ forms and nitrates catalytic tyrosines on key enzymes and can inactivate peroxiredoxins, collapsing front-line H_2_O_2_ buffering. In parallel, iron dyshomeostasis—expansion of the labile iron pool and damage to Fe–S clusters—lowers the threshold for ferroptosis-like lipid peroxidation in PUFA-rich neuronal membranes, particularly in regions with heavy metabolic demand (Liu et al. 2019).

For translation, multi-parameter panels that combine CSF/serum lipid-peroxidation markers (e.g., F_2_-isoprostanes, HNE adducts), protein injury footprints (nitrotyrosine, carbonyls) and compartment-targeted reporters in experimental models (matrix/ER/nuclear H_2_O_2_ sensors, peroxiredoxin redox state) and that are aligned to cognitive or motor end-points allow mechanism-anchored evaluation of interventions (Murphy et al. 2022b).

Such panels can test NOX inhibition (isoform-specific), mitochondrial modulators (improving biogenesis/mitophagy, tempering RET and Ca²⁺ overload), iron management (chelators, ferritinophagy restraint) and RSS augmentation (controlled H_2_S/persulfide donors) designed to preserve physiological H_2_O_2_ signalling needed for plasticity while reducing nitrative and Fenton chemistry that drives irreversible injury (Li et al. 2025).

### Cancer: dual roles in initiation, progression and therapy response

In cancer, ROS are Janus-faced: during initiation, oxidative DNA lesions (e.g., 8-oxoG, single-/double-strand breaks) and lipid-derived electrophiles from peroxidation increase mutational burden and clonal selection (O’Reilly et al. 2024). During progression, NOX- and mitochondria-derived H_2_O_2_ sustain proliferative signalling by reversibly oxidizing protein tyrosine phosphatases (PTPs), lower thresholds for EMT and promote invasion and immune evasion via redox-tuned cytokine circuits and ECM remodelling (Nigam et al. 2025).

Tumors simultaneously rewire antioxidants—activating NRF2 and elevating GSH/thioredoxin systems—creating a narrow therapeutic window in which pushing ROS beyond buffering capacity triggers apoptosis or ferroptosis, whereas indiscriminate scavenging blunts therapy and fosters resistance (Reczek and Chandel 2017).

Mechanism-informed strategies therefore target sources and nodes rather than applying blanket antioxidants: source-targeted ROS amplification inside tumors (e.g., radiotherapy or certain chemotherapies combined with ferroptosis inducers and microenvironmental iron loading to accelerate lipid peroxidation) can overwhelm malignant buffers, while node inhibition (selective NOX isoforms, FSP1/CoQ rescue circuits, or lipid-peroxidation repair pathways) can re-sensitize resistant clones (Jiang et al. 2020a).

Treatment response should be tracked with redox proteomics (cysteine-switch/oxidized-PTP signatures), peroxiredoxin redox states (as sentinels of H_2_O_2_ flux) and lipid-peroxide markers (F_2_-isoprostanes, oxidized phospholipids), enabling adaptive dosing that preserves beneficial redox signalling in normal tissues while exploiting malignant redox liabilities (Kisty et al. 2023).

### Cross-cutting themes and clinical translation

Across cardiovascular, metabolic, neurodegenerative and oncologic disorders, a common logic governs redox pathology: where ROS are generated (source and subcellular compartment) determines which molecular targets and nearby antioxidant buffers they encounter. When they arise as brief pulses versus sustained plateaus separates adaptive signalling from cumulative injury and how much flux flows sets the thresholds for ONOO^−^ formation, Fenton ·OH production and ferroptosis ignition (Selvaraj et al. 2025).

For clinical translation, this argues for multi-parameter panels that pair species-level reporters (e.g., DHE-HPLC to quantify O_2_·− via 2-OH-E⁺. Compartment-targeted HyPer to track H_2_O_2_ in matrix/ER/nucleus) with damage footprints (F_2_-isoprostanes, nitrotyrosine, protein carbonyls, 8-oxoG) and iron metrics (calcein-quench assays of the labile iron pool with chelator validation, ferritin, HO-1 and ferritinophagy markers) and that are interpreted with strict source attribution and compartment awareness (Murphy et al. 2022b) (as outlined in “Methods for ROS detection: techniques, limitations and pitfalls” section).

Therapeutically, node-specific, compartment-aware interventions—tuning NOX/NOS activity, reinforcing mitochondrial quality control, alleviating ER stress, managing iron and suppressing ferroptosis when indicated and providing calibrated RSS support—consistently outperform blanket antioxidant scavenging by preserving beneficial H_2_O_2_-based signalling while preventing convergence onto damaging chemistries such as peroxynitrite formation, Fenton-driven hydroxyl radicals and runaway lipid peroxidation (Li et al. 2025).

## Therapeutic strategies targeting ROS: pharmacological and lifestyle interventions

Effective ROS control is less about “soaking up oxidants” and more about modulating the nodes and compartments where reactive fluxes are generated, propagated and decoded (Sun et al. 2020). This section organizes interventions by source/compartment, then integrates cross-cutting network targets and lifestyle levers, always aligning with the manuscript’s organizing rule—where, when and how much. We draw directly on the mechanistic groundings and pitfalls outlined in “Biological Sources and Subcellular Compartments of ROS–Crosstalk with RNS/RSS and iron” sections and the detection standards in “Methods for ROS detection: techniques, limitations and pitfalls” section.

### First principles for therapy design

Therapy design should follow three first principles: target the source before the sink, prioritizing isoform- or site-specific modulation of NOX enzymes, mitochondrial ROS sites, ER oxidoreductases, or iron handling rather than indiscriminate scavenging that can blunt physiological signalling (Sies et al. 2022).

Treat by compartment, matching drugs and delivery to the exact locale—mitochondrial matrix or IMS, ER lumen, peroxisome, or plasma-membrane microdomains—where the reactive chemistry originates and use biomarkers to time and titrate (Giacomello et al. 2020), pairing species-level reporters with damage footprints and iron metrics (“Methods for ROS detection: techniques, limitations and pitfalls” section) to decide when to escalate, switch, or de-escalate therapy so that beneficial H_2_O_2_ signalling is preserved while pathological fluxes are curtailed.

### Source- and compartment-targeted Pharmacology

Before drilling into each therapeutic node, it helps to set the practical frame that will guide selection and monitoring: (1) prioritize source identity and subcellular compartment over indiscriminate “ROS scavenging,” following the where–when–how much rule; (2) use triangulated biomarkers that pair species/compartment readouts (e.g., DHE→2-OH-E⁺ by HPLC for O_2_·−, compartment-targeted HyPer sensors, and peroxiredoxin redox state) with damage footprints (F_2_-isoprostanes, carbonyls/nitrotyrosine, 8-oxoG) and iron/ferroptosis indices (labile iron pool, ferritin/HO-1/NCOA4); and (3) tune dose, timing, and delivery (mitochondria-, ER-, or plasma-membrane-targeted) to preserve physiological H_2_O_2_ signaling while attenuating pathological microdomains. With this in mind, the following subsections address targets by source/compartment, starting with NADPH oxidases (NOX/DUOX), whose nuanced control can modulate redox tone without compromising host defense, Table 1.

**Table 1:** Phase-specific ROS-guided therapy (acute, subacute, chronic): goals, dominant sources, key interventions and biomarkers

Block	Key question	Action	Readouts (examples)
Inputs	Where? dominant source & compartment	Map source/compartment	Targeted sensors (e.g., HyPer), DHE-HPLC, Prx redox
	When? pulse vs. tone	Place in phase (acute → chronic)	Time course of markers
	How much? oxidant load	Set safe set-point	Damage panel (F2-isoprostanes, carbonyls)
Decision	What to target first?	Pick source & compartment	–
Dosing rules	How to intervene?	Source control > bulk antioxidants; treat by compartment; titrate to biomarkers	Preserve physiological H_2_O_2_, avoid ONOO^−^/·OH
Phases	Timing of therapy	Acute (0–24 h) dampen bursts → Subacute (D2–14) restore set-point → Chronic (≥ W3) light maintenance	Gate by improvement in markers/function

Node/compartment	Main action	Typical readouts
NOX/DUOX (PM microdomains/endosome)	Isoform/location-selective attenuation; co-target pathways	DHE-HPLC (2-OH-E⁺), localized HyPer, FMD
Mitochondria (RET, ΔΨm, Ca²⁺)	Limit RET/hyperpolarization; QC (biogenesis/mitophagy); tune MAM Ca²⁺	Mito-HyPer (matrix/IMS), Prx3 redox, function
ER/Secretory (Ero1–PDI)	Modulate—not suppress—UPR; support Prx4/GPx7/8; tune MAMs	HyPer-ER/roGFP-Orp1, BiP/CHOP/XBP1s
Peroxisome—Mito	Dose β-oxidation (PPARα); sustain catalase/Prx5	PTS1-targeted sensors; lipid-ox LC-MS
Iron/Ferroptosis (LIP, GPX4)	Iron hygiene; support GPX4/Se; RTAs; curb ferritinophagy	LIP de-quench, oxidized PLs, F2-isoprostanes
RNS/RSS	Re-couple NOS (BH4/Arg); controlled H_2_S/polysulfides	NO bioavailability/FMD, nitrotyrosine, persulfidation

#### NADPH oxidases (NOX/DUOX)

For NADPH oxidases (NOX/DUOX), the rationale is to curb pathological microdomain activity—from NOX1/2/4/5 and DUOX—that drives endothelial dysfunction, fibrosis and innate immune priming, while preserving the tightly tuned H_2_O_2_ pulses required for physiological signalling, hence selective attenuation (by isoform and locale) is preferred over global blockade (Altenhöfer et al. 2015).

The therapeutic approach centers on isoform-selective inhibitors or genetic silencing, complemented by pathway co-targets that feed these enzymes (e.g., angiotensin/PKC–NOX axes) and precision delivery to the relevant plasma-membrane, endosomal, or nuclear microdomain to avoid off-target suppression of host defense (Cipriano et al. 2023a).

Readouts should triangulate source and compartment: quantify superoxide with DHE-HPLC (particularly informative for NOX2), track H_2_O_2_ with compartment-targeted HyPer sensors and couple these to endothelial function tests (e.g., flow-mediated dilation or wire myography), while avoiding naïve reliance on raw probe fluorescence (see “Small-molecule fluorescent/chemiluminescent probes” section) that cannot disambiguate species or sources (Pak et al. 2020; Zielonka et al. 2019).

#### Mitochondrial redox control

For mitochondrial redox control, the rationale is that reverse electron transport (RET) at complex I, sustained elevation of ΔΨm, Ca²⁺ overload and high-flux activity at the I_Q/III_Qo sites generate matrix and intermembrane-space ROS bursts that flip physiological signalling into damage during stress states such as ischemia–reperfusion (Gibbs et al. 2023).

Accordingly, the approach is to fine-tune substrate entry and ΔΨm (e.g., adjust succinate/fatty-acid flux, avoid excessive hyperpolarization), limit RET in high-risk windows, reinforce mitochondrial quality control (biogenesis and mitophagy to cull ROS-prone organelles) and buffer Ca²⁺ transfer at MAMs to prevent feed-forward ROS/Ca²⁺ loops—favoring these node- and compartment-specific levers over blanket antioxidant suppression (Brand et al. 2016).

Readouts should triangulate source and compartment by using matrix/IMS-targeted reporters for H_2_O_2_ dynamics, peroxiredoxin-3 (Prx3) redox state as a fast sentinel of matrix oxidant load and functional recovery after stress (e.g., respiration, contractility, cell survival) to confirm that interventions restore signalling–damage balance rather than merely lower bulk ROS (Cardozo et al. 2023; Koren et al. 2023).

#### ER redox and proteostasis

For ER redox and proteostasis control, the rationale is that Ero1–PDI oxidative folding cycles intentionally produce H_2_O_2_ in the ER lumen. When secretory load rises or the UPR^ER is engaged, excess luminal oxidant can spill over into the cytosol/mitochondria via MAMs, propagating Ca²⁺ dysregulation and cell-wide stress. Accordingly, the therapeutic approach is to titrate—not suppress—the UPR^ER, using chemical chaperones and selective PERK/IRE1 modulators to reduce misfolded-protein pressure while preserving adaptive signalling (Feldman et al. 2021; Zito 2015).

To reinforce ER-localized peroxidases (Prx4, GPx7/8) that detoxify and relay H_2_O_2_ within the lumen rather than applying bulk antioxidants that erase physiological cues and to modulate Ca²⁺ transfer at MAMs so redox and calcium do not amplify each other (Kanemura et al. 2020).

Readouts should align with this compartment logic: deploy HyPer-ER or roGFP-Orp1 reporters to capture ER-confined H_2_O_2_ dynamics (avoiding cytosolic averaging), track ER stress markers (e.g., BiP/GRP78, CHOP, spliced XBP1) to ensure adaptive, not terminal, UPR engagement and quantify downstream metabolic resilience (protein secretion fidelity, ATP/redox balance, sensitivity to secondary hits) to verify that interventions restore ER redox homeostasis and limit maladaptive crosstalk to mitochondria (Melo et al. 2017).

#### Peroxisome–mitochondria axis

For the peroxisome–mitochondria axis, the rationale is that peroxisomal β-oxidation is a major H_2_O_2_ source (via acyl-CoA oxidases and other flavoproteins) and peroxiporins allow H_2_O_2_ to exit the organelle and coordinate with cytosolic and mitochondrial pathways—so when flux outstrips buffering, lipid-peroxide pressure rises and spills into neighbouring compartments (Lismont et al. 2019, 2022).

Accordingly, the approach is to tune PPARα-driven β-oxidation surges (dietary/farmacologic cues) to actual disposal capacity, ensure luminal detox is not the bottleneck by maintaining catalase and Prx5 activity and route fatty acids intelligently—steering chain length and saturation toward mitochondria only when respiratory capacity and antioxidant buffers can keep pace, thereby avoiding peroxisome-to-mitochondria mismatch that propagates peroxidation (Tahri-Joutey et al. 2021).

Readouts should reflect this compartment logic, combining peroxisome-targeted H_2_O_2_ reporters (e.g., HyPer/roGFP-Orp1 with PTS1 “SKL” tagging) to track local oxidant dynamics with LC–MS lipid-peroxidation markers (F_2_-isoprostanes, oxidized phospholipids, HNE adducts) to quantify downstream burden and confirm that interventions reduce peroxide leakage while preserving necessary peroxisomal metabolism (Arnaud et al. 2023; Dushianthan and Postle 2021).

#### Iron and ferroptosis control

For iron and ferroptosis control, the rationale is that the labile iron pool (LIP) catalyzes H_2_O_2_→·OH via Fenton/Haber–Weiss chemistry and, when PUFA-containing phospholipids (PUFA-PLs), NOX/LOX activities and GPX4 insufficiency coincide, licenses ferroptosis—a lipid peroxidation–driven cell death pathway (Jiang et al. 2021).

Accordingly, the therapeutic approach is to lower catalytic iron and interrupt chain propagation by combining chelation (e.g., deferoxamine or deferiprone) with restraint of ferritinophagy (NCOA4) to curb Fe²⁺ release, managing HO-1 induction so heme breakdown does not overexpand LIP, restoring GPX4 capacity via selenium sufficiency and GSH support and deploying radical-trapping antioxidants (ferrostatin-1, liproxstatin-1) to terminate lipid-radical chains. In selected contexts, modulate ACSL4/LPCAT3 (PUFA loading into membranes) and LOX activity to reduce peroxidation substrates and enzymatic initiation, while reinforcing parallel anti-ferroptotic circuits—FSP1–CoQ at the plasma membrane and DHODH–CoQH_2_ in mitochondria—to buffer lipid radicals without abolishing physiological signalling (Mao et al. 2021; Zhou et al. 2024a).

Readouts should verify both mechanism and effect: quantify LIP with calcein-quench assays and demonstrate chelator reversibility, track ferritin, HO-1 and NCOA4 to index iron handling and measure lipid-peroxide signatures (oxidized phospholipids, F_2_-isoprostanes, HNE adducts) alongside functional endpoints, ensuring that interventions reduce Fenton hotspots and ferroptotic drive while preserving necessary iron-dependent metabolism (Hider et al. 2023; Oskolkova et al. 2022).

#### RNS/RSS network nodes

For RNS/RSS network modulation, three levers are complementary and should be applied with dose- and compartment-awareness: NOS re-coupling aims to restore nitric-oxide production and suppress superoxide leak by supporting BH₄ availability (limiting oxidative BH₄→BH_2_ loss), ensuring adequate L-arginine flux (and arginine: ADMA ratio) and reducing ADMA accumulation (e.g., improving DDAH activity), thereby shifting uncoupled NOS back to NO synthesis and lowering ONOO^−^ formation (Łuczak et al. 2020).

GSNOR/denitrosylation tuning seeks to rebalance reversible S-nitrosation—a bona fide signalling modification—without collapsing NO pathways, using context-specific modulation of GSNOR and denitrosylating systems (thioredoxin/γ-glutamyl transpeptidase axes) so that excessive, persistent S-nitrosation is relieved while physiological, transient nitrosation on target cysteines is preserved (Chatterji and Sengupta 2021; Gusarov and Nudler 2018).

RSS augmentation leverages controlled-release H_2_S/persulfide donors (including organelle-targeted chemotypes when appropriate) to stabilize redox-sensitive thiols via persulfidation and to recycle sulfenylated switches back to the signalling-competent state—dosed to local set-points to avoid off-target mitochondrial inhibition, hypotension, or interference with immune defence and guided by biomarkers such as nitrosothiols/persulfide load, peroxiredoxin redox state and compartment-targeted H_2_O_2_ reporters to verify that NO and sulfur signalling are restored while nitrative and Fenton-linked chemistries are constrained (Filipovic et al. 2018; Magierowska et al. 2022).

### Network-level modulators and why “antioxidant loading” underperforms

For network-level modulation, leverage but don’t overdrive endogenous defences: tuning the NRF2/Keap1 (Cuadrado et al. 2019) axis can replenish glutathione (GSH), thioredoxin/peroxiredoxin (Trx/Prx) systems and phase-II detox enzymes when buffers are depleted, yet indiscriminate or chronic activation risks tumor tolerance and may blunt beneficial hormetic signalling, so deploy NRF2 agonism in context-specific windows with biomarker guidance (e.g., GSH/GSSG, Prx redox state) (Dodson et al. 2019).

In parallel, directly supporting the GSH/Trx systems—via precursors (cysteine/N-acetylcysteine, selenium for selenoenzymes), enzyme cofactor support, or metabolic rewiring—restores redox buffering and helps the proteome re-enter the “reversible cysteine” zone (see “The cysteine “oxidation code”” section), preserving physiological H_2_O_2_-mediated signalling while preventing spillover into irreversible oxidation (Ren et al. 2017).

By contrast, megadose, non-targeted antioxidants (blanket vitamins or generic scavengers) typically underperform because they ignore source identity and compartment, pool signals much like bulk Amplex-style readouts and suppress adaptive redox cues essential for resilience and recovery—hence the preference for node- and compartment-aware strategies that are titrated to objective redox biomarkers rather than applied indiscriminately (Le Gal et al. 2015).

### Lifestyle interventions that reshape ROS tone (mechanism-anchored)

Lifestyle levers can reset ROS tone in ways drugs often can’t: exercise training induces mitochondrial biogenesis and mitophagy, improves endothelial NO bioavailability and upregulates endogenous antioxidant systems so that the pulsatile ROS generated during bouts acts as a mitohormetic signal rather than a damaging load (Guan et al. 2019).

Nutritional strategies that favor caloric moderation and healthier fat profiles lower ER secretory stress, reduce peroxisome–mitochondria mismatch and diminish LIP-driven Fenton risk, while ensuring selenium sufficiency supports GPX4 and ferroptosis control (Matai et al. 2019).

Sleep and circadian alignment stabilize metabolic and redox rhythms that gate ROS detoxification and macromolecular repair, improving resilience to daytime oxidative challenges and environmental hygiene—limiting UV/photosensitization in exposed tissues and managing metal exposures that expand the labile iron pool—curbs non-enzymatic ROS generation and Fenton chemistry at their source (Davinelli et al. 2024).

These interventions should be individualized and biomarker-guided (e.g., endothelial function, Prx redox state, GSH/GSSG, F_2_-isoprostanes, LIP assays) and titrated to preserve beneficial H_2_O_2_-based signalling while preventing convergence onto ONOO^−^, hydroxyl-radical formation and runaway lipid peroxidation (Frijhoff et al. 2015; Koike et al. 2022).

### Implementation: a biomarker-guided, compartment-aware algorithm

Implement a biomarker-guided, compartment-aware algorithm: first, phenotype the redox problem with a multi-parameter panel that combines species reporters (DHE-HPLC to quantify O_2_·− via 2-OH-E⁺, compartment-targeted HyPer to track H_2_O_2_) (Meier et al. 2019)with damage footprints (F_2_-isoprostanes, nitrotyrosine, protein carbonyls, 8-oxoG) and iron metrics (calcein-quench assays of the labile iron pool with chelator validation, plus ferritin/HO-1/ferritinophagy markers) (Riedelberger and Kuchler 2020).

Second, assign the source and compartment—distinguish NOX versus mitochondrial versus ER/peroxisomal origins and map the locale (matrix, IMS, ER lumen, plasma-membrane microdomain)—and define the temporal pattern (brief pulses vs. sustained plateaus) that separates signalling from injury (Gianazza et al. 2021).

Third, select node-specific therapy tailored to that map (e.g., NOX isoform modulation, RET tempering and mitochondrial quality control, UPR^ER tuning, iron/ferroptosis control, NOS re-coupling, RSS support) and co-prescribe lifestyle measures (structured exercise, nutrition, sleep/circadian alignment and exposure control) that reset redox tone without erasing adaptive cues (Meng et al. 2021).

Finally, titrate to biomarkers and function—not just symptoms—aiming to preserve physiological H_2_O_2_-based signalling while preventing convergence onto peroxynitrite (ONOO^−^) formation, Fenton-derived ·OH and runaway lipid peroxidation (Sies 2021).

### Safety, trade-offs and trial design

Avoid trading one pathology for another: excessive suppression of ROS can blunt host defense, neuroplasticity and adaptive stress responses, whereas excessive amplification can select resistant cancer clones or precipitate ferroptosis in vulnerable, PUFA-rich tissues—so aim for balance, not extremes (Kakizawa et al. 2024).

Use adaptive dosing and staging matched to disease phase and compartment: for example, early ischemia–reperfusion may benefit from acute, time-boxed control of mitochondrial bursts and labile iron to limit Fenton chemistry, while chronic vascular or organ remodeling calls for rebalancing NOX activity and ER stress together with lifestyle re-entrainment (exercise, nutrition, sleep, exposure control) to reset redox tone without erasing physiological H_2_O_2_ signalling (Poznyak et al. 2020; Sawicki et al. 2023).

Finally, report with rigor so decisions are reproducible and mechanism-anchored: adhere to “Methods for ROS detection: techniques, limitations and pitfalls” section standards—include specificity controls (SOD/catalase, chelators), calibrations and kinetic context and compartment targeting—so that therapeutic readouts map cleanly to species, source and location, allowing precise titration rather than blanket scavenging or untargeted escalation.

## Role of ROS in transcription regulation

Transcription is shaped by where and how oxidants reach DNA and the transcriptional machinery. Cells generate localized ROS near the nuclear envelope (NOX isoforms), the outer mitochondrial membrane (MAO facing the cytosol) and cytosolic dehydrogenase complexes adjoining the nucleus (Kaludercic et al. 2023; Moloney et al. 2017).

O_2_·− and its product H_2_O_2_ dominate, while ultra-local ·OH arises at metal centers within chromatin (Attar et al. 2020). Peroxiporin channels and redox relays funnel H_2_O_2_ into defined nuclear microdomains, so targets experience short-range gradients rather than bulk oxidative stress. This spatial logic explains how reversible cysteine oxidation can modulate transcription with selectivity while limiting collateral DNA damage (Talwar et al. 2020).

### The “cysteine code” in transcriptional control

Most transcriptional regulators contain redox-sensitive cysteines that cycle through sulfenylation (Cys–SOH), disulfide or S-glutathionylation adducts (Matsui et al. 2020) and—under harsher conditions—irreversible sulfinylation/sulfonylation. These modifications tune DNA binding, cofactor docking, nuclear import/export and turnover (Wang et al. 2020).

In parallel, persulfidation (Cys–SSH) from reactive sulfur species functions as a rapid, reversible rheostat that stabilizes catalytic thiols and can “rescue” sulfenic intermediates back to signaling-competent states, preserving transcriptional responsiveness while preventing over-oxidation. Together, these cycles form a chemical alphabet that encodes amplitude and duration of ROS cues into gene expression programs (Dóka et al. 2020).

### Sentinel pathways and factors

NRF2/Keap1. Keap1’s sensor cysteines (e.g., Cys151/273/288) register electrophiles and oxidants (Saito et al. 2016). Their reversible modification disrupts Keap1-mediated NRF2 ubiquitination, allowing NRF2 to enter the nucleus and bind AREs to induce antioxidant and detox genes (HO-1, NQO1). Persulfidation of Keap1 provides a fast, reversible route to NRF2 release, whereas irreversible S-alkylation by electrophiles yields prolonged activation but at the cost of thiol depletion—two modes with different kinetic and safety profiles (Xie et al. 2016).

NF-κB. The canonical axis is governed by redox-tunable cysteines in IKKβ (Cys179) and in the DNA-binding cores of p65 (Cys38) and p50 (Cys62). Persulfidation operates as a high-speed volume control across this cascade, while stronger electrophilic hits can impose longer-lived off states. In inflammatory milieus rich in ROS/RNS, the balance between these chemistries determines cytokine output and chromatin residence (Wilkinson and Gow 2021; Zhang et al. 2019b).

AP-1, HIF-1α and stress pathways. O_2_·− pulses sufficient to oxidize regulatory thiols activate AP-1 and HIF-1α (Andrés et al. 2025b), promoting stress-response and metabolic reprogramming. Sustained O_2_·−/ONOO^−^ plateaus push the system toward damage signalling and mitochondrial failure. Thus, the same oxidant, delivered with different timing and intensity, flips transcription from adaptation to pathology (Manuelli et al. 2022).

### Chromatin as a redox sensor

Redox events regulate chromatin at multiple levels. Histone-modifying enzymes (HATs/HDACs, demethylases) harbor catalytic cysteines and Fe–S or non-heme iron cofactors vulnerable to oxidation, transiently altering activity and locus accessibility. Oxidative DNA adducts such as 8-oxoG are not only lesions but also contextual signals that recruit base-excision machinery (e.g., OGG1) and remodel local chromatin, thereby coupling genome surveillance to transcriptional tuning. When repair is overwhelmed or mis-targeted, however, strand breaks and PARP over-activation derail transcription and bioenergetics (Lamadema et al. 2019; Pan et al. 2023).

### Integration with RNS and RSS

Cross-talk with RNS and RSS adds layers of control. NOS–NOX reciprocity and ONOO^−^ formation shape nitrative footprints (e.g., 3-nitrotyrosine) that reprogram transcription factor activity. In parallel, H_2_S/persulfides augment transcriptional fidelity by persulfidating sensor cysteines (Keap1, NF-κB) and maintaining the reversible zone of the cysteine code (Andrés et al. 2024a). NRF2 in turn upregulates trans-sulfuration enzymes (CSE/3-MST), creating a protective feed-forward loop (Campolo et al. 2020). These axes collectively decide whether redox inputs resolve as adaptive gene expression or as inflammatory and death-pathway transcription (Jamaluddin et al. 2022).

### Quantitative rules: “where, when, how much”

Transcriptional outputs are dictated by source/compartment, pulse design and dose. Brief, low-amplitude H_2_O_2_ pulses delivered to nuclear microdomains favor reversible cysteine switches on TFs and cofactors (Wernet et al. 2015).

Sustained or repeated fluxes saturate local peroxiredoxins and glutathione pools, spill into iron-rich chromatin niches and bias toward ONOO^−^ and Fenton chemistry that disrupt transcription. This framework recurs across tissues and diseases, explaining why the same factor (e.g., NRF2 or NF-κB) can mediate resilience in one context and pathology in another (Calabrese et al. 2019; Itkin and Rafii 2017).

### How to measure transcription-linked redox with rigor

Methodologically, pair protein-centric readouts (redox proteomics of TF cysteines-Prx redox state) with compartment-targeted H_2_O_2_ reporters in the nucleus or inner-nuclear membrane to capture flux and map footprints (3-nitrotyrosine, 8-oxoG) to link exposure with functional outcomes (Vaissier and Van Voorhis 2017; Zhang and Bar-Peled 2023).

Use genetic/pharmacologic separation (NOX/NOS isoforms, CSE/3-MST, iron chelation) to assign chemistry and report calibration, matrix and kinetics to distinguish fast signalling from damage plateaus. These standards prevent conflating cause and consequence in transcriptional redox biology (Altenhöfer et al. 2015; Winterbourn 2020).

### Therapeutic angles: modulate nodes, preserve signalling

Because transcriptional regulation lives in the reversible zone of the cysteine code, interventions should modulate nodes instead of globally scavenging oxidants. Practical levers include Keap1/NRF2 tuning with attention to electrophile vs. persulfide kinetics, isoform- and microdomain-specific NOX control to shape nuclear H_2_O_2_ pulses, NOS re-coupling to limit ONOO^−^ and sulfur-axis support to maintain persulfidation capacity. Biomarker-guided dosing (e.g., TF cysteine redox state, Prx cycling, 8-oxoG with repair indices) helps preserve beneficial transcriptional cues while preventing convergence onto nitrative and Fenton chemistry (Meng et al. 2021; Rorsman and Ashcroft 2018).

## Biomarkers of ROS and clinical translation: from bench to bedside

Clinically useful redox biomarkers should (1) reflect a defined chemistry (e.g., O_2_·− vs. H_2_O_2_ vs. nitration/lipid peroxidation footprints), (2) report from the right compartment or tissue and (3) change with mechanism-based therapy. In practice, single readouts underperform. Multi-parameter panels that pair species-level reporters with damage footprints and iron metrics provide more reliable attribution and stratification (Chen et al. 2017; Collins et al. 2015). This panel logic underpins our framework across “Methods for ROS detection: techniques, limitations and pitfalls”–Therapeutic strategies targeting ROS: pharmacological and lifestyle interventions” section and is the bedrock for translation.

### What to measure: a practical menu

A clinically useful redox panel should integrate four complementary layers into a single, interpretable picture: species-level reporters (mechanistic layer)—use DHE-HPLC to quantify O_2_·− specifically via its diagnostic 2-OH-E⁺ product (Michalski et al. 2020) (never raw fluorescence) and deploy compartment-targeted HyPer variants to monitor H_2_O_2_ in the mitochondrial matrix, ER lumen, or nucleus (Aki et al. 2023).

Pair these with selective NO/ONOO^−^ probes applied under kinetic and scavenger/enzyme controls so they define flux and location rather than a pooled “ROS” signal (Zhou et al. 2024b). Damage footprints (integrative layer) should track F_2_-isoprostanes/oxidized phospholipids, protein carbonyls/3-nitrotyrosine and 8-oxoG/strand breaks, confirming by LC–MS where feasible to avoid colorimetric artifacts and explicitly linking each footprint back to its source and compartment using the species layer above (Khoury et al. 2018).

Iron metrics (propensity layer) must quantify the labile iron pool (LIP) by calcein-quench assays with chelator reversibility and include ferritin, HO-1 and NCOA4 (ferritinophagy) to index iron handling, thereby gauging the likelihood of Fenton ·OH formation and ferroptosis (Le et al. 2024).

Finally, protein-centric redox sentinels add temporal resolution: monitor peroxiredoxin (Prx) redox state (non-reducing gels/redox Westerns) as a near-real-time readout of H_2_O_2_ load and buffer saturation and use cysteine-redox proteomics to map reversible thiol switches within the very pathways you intend to modulate (Stretton et al. 2020). Together—and only when interpreted with proper controls, calibration and compartment awareness—these layers provide a validated, decision-ready view of species, source, location and risk.

### Matrices, pre-analytics and reporting

Specify matrix (plasma, serum, urine, CSF, exhaled breath condensate, tissue), turnaround, LOD and quality controls. Pre-analytic handling (time to spin, storage, light/pH/temperature) can swamp true biology (Lippi et al. 2015). Bedside or minimally invasive POC modalities are useful for trajectory monitoring and triage, but they rarely resolve species or compartments and should be anchored to lab-grade assays for mechanism (Khan et al. 2024).

### Disease-anchored panels and how to act on them

For disease-anchored panels, tailor the readouts to the biology and act on the map they provide: in cardiovascular/vascular beds, pair DHE-HPLC (to quantify O_2_·−) with compartment-targeted HyPer for H_2_O_2_, then add F_2_-isoprostanes/3-nitrotyrosine and iron indices to distinguish NOX-driven endothelial dysfunction (O_2_·−→ONOO^−^ lowering NO bioavailability) from mitochondrial bursts typical of ischemia–reperfusion (Bækgaard Nielsen et al. 2017).

Use this attribution to choose NOX isoform modulation, mitochondrial conditioning and iron management (Kračun et al. 2025). In metabolic liver/adipose, overlay ER-/peroxisome-targeted H_2_O_2_ reporters on lipid-peroxide panels and LIP assays to separate ER load/peroxisomal β-oxidation from mitochondrial mismatch, guiding UPR^ER tuning, PPARα pacing and ferroptosis-risk control (Lismont et al. 2022).

For neurodegeneration, combine CSF F_2_-isoprostanes/HNE adducts, nitrotyrosine/carbonyls and model-based compartment reporters aligned to cognitive/motor endpoints to evaluate NOX inhibition, mitochondrial quality control, iron restraint and RSS augmentation, preserving the physiological H_2_O_2_ signalling required for plasticity (Ahmad et al. 2024; Calvo-Rodriguez et al. 2024).

In oncology, integrate redox proteomics (PTP/cysteine-switch signatures), peroxiredoxin redox states and lipid-peroxide markers to time source-targeted ROS amplification (e.g., radiotherapy/chemotherapy plus ferroptosis inducers) versus node inhibition (NOX, FSP1/CoQ circuits) (Chen et al. 2023b). Such panels support adaptive dosing that exploits malignant redox liabilities while sparing normal-tissue signals and avoiding indiscriminate scavenging.

### Bedside translation: superoxide-guided care in critical illness (worked example)

In the ICU, serial O_2_·− measurement has shown prognostic and actionable value across sepsis, TBI and post-cardiac surgery cohorts. Algorithms that gate therapy on O_2_·− thresholds (e.g., early NOX2 inhibition, escalation to mitochondria-targeted agents and only then bulk antioxidants) exemplify time-stamped, biomarker-guided escalation–de-escalation (Colombi et al. 1969; Thondapu et al. 2021). This approach operationalizes “where, when, how much” at the bedside and provides a reproducible scaffold for trials.

### From panel to practice: a stepwise implementation pathway

Begin by defining the clinical decision—risk stratification, target engagement, or response-guided dosing—and then build a minimal effective panel that links species → footprint → propensity (iron) so each measurement serves that decision. Next, anchor point-of-care (POC) trends to lab-grade assays during initial validation to confirm specificity and calibration. Third, pre-register analysis plans (normalization per protein/cell or tissue mass, oxygenation status, ΔΨm adjustments for mito-probes) and the full set of specificity controls (SOD/catalase for O_2_·−/H_2_O_2_, chelators for Fenton dependence, genetic knockdown/knockout for source attribution) to prevent misassignment (Bossuyt et al. 2015; Kronenfeld et al. 2015).

Finally, pair biomarkers with microdomain-aware interventions—tuning NOX/NOS activity, tempering RET and supporting mitochondrial quality control, modulating UPR^ER, controlling iron/ferroptosis and augmenting RSS where appropriate—while co-prescribing lifestyle levers (structured exercise, nutrition and circadian hygiene) so that therapy is targeted to the where, when and how much of the redox problem and is titrated to objective readouts rather than symptoms alone (Chen et al. 2023a; Forman and Zhang 2021).

### Validation standards for clinical readiness

Report calibration curves, raw chromatograms/spectra, instrument settings and time-stamped protocols. Where possible, include rescue/chelator reversibility (e.g., LIP by calcein de-quench) to prove on-target signal. Trials should co-primary (1) mechanistic change (e.g., Prx cycling, 2-OH-E⁺, LIP) and (2) clinical improvement (function, organ scores), preventing the common trap of “ROS lowering without benefit” (Guideline 2022).

### What’s next

Priorities include: robust reference ranges by matrix and disease stage, automated analytics that integrate species/footprint/iron layers, contact-site imaging to map microdomain sources and adaptive platform trials where biomarker gates determine entry and dose for node-specific, compartment-aware therapies (Woodcock and LaVange 2017). These steps will convert redox panels from research tools into routine companions for precision therapeutics.

## Therapeutic perspectives

We translate the core rules of ROS biology—source, compartment and pulse kinetics—into practical therapy. Rather than blanket antioxidant loading, we focus on node- and compartment-targeted interventions (NOX/NOS modulation, mitochondrial control, ER redox, iron/ferroptosis) that preserve physiological H_2_O_2_ signalling while preventing peroxynitrite, Fenton ·OH and lipid peroxidation (Rosenbaum et al. 2020). We overview the clinical landscape, outline rational combinations with classic antioxidants, define analytics that measure the right species in the right place/time and propose phase-sequenced regimens guided by biomarkers.

### Clinical trials and the translational landscape of ROS-Targeted strategies

Clinical translation of ROS control aims to restore the redox set-point without abolishing physiological H_2_O_2_ and O_2_·− signaling. In practice, approaches cluster into four fronts that span distinct compartments and time scales:

#### Inhibitors of ROS sources

NOX (NADPH oxidases): To curb pathological ROS, two complementary strategies are often combined. NOX inhibition targets NADPH oxidases at the plasma membrane and within organelles: using selective NOX1/4 blockers or broader pan-NOX agents can dampen site-specific ROS generation, which in turn may slow fibrotic remodeling, protect endothelial function and vascular tone, and ease downstream metabolic stress. Mitochondrial modulation aims to prevent burst-like ROS surges, especially during ischemia–reperfusion: a modest degree of uncoupling lowers the proton motive force so the respiratory chain is less prone to leak electrons (Hartmann et al. 2018; Invernizzi et al. 2023).

Restricting substrate influx (e.g., fatty acids or succinate) avoids over-reducing the electron carriers at the critical moment of reperfusion, and specifically blunting reverse electron transport into Complex I limits the high-amplitude ROS spikes that drive acute injury and propagate inflammatory signaling. Together, these interventions reduce ROS at both extramitochondrial and mitochondrial sources, addressing the timing, location, and intensity of oxidative stress (Yamada et al. 2020).

#### Scavengers and site-directed mimetics

Mitochondria-focused antioxidant strategies concentrate defenses where ROS are made. Lipophilic TPP⁺ conjugates accumulate across the inner membrane into the matrix, delivering antioxidant payloads directly to mitochondrial sites and avoiding systemic dilution (Jiang et al. 2020b). In parallel, SOD and catalase mimetics act as enzymatic stand-ins: SOD mimetics rapidly dismutate superoxide to H_2_O_2_, and catalase-like agents decompose that H_2_O_2_ to water and oxygen, limiting diffusion-driven damage (Grujicic and Allen 2024).

Downstream, GPx mimetics and the endogenous peroxiredoxin/thioredoxin (Prx/Trx) cycle clear residual H_2_O_2_ with high efficiency while maintaining the redox “tuning” needed for physiology—allowing reversible cysteine oxidations that encode normal signaling, yet preventing the sustained or widespread oxidation that tips cells into dysfunction. Together, these tools localize antioxidant activity, shape ROS flux rather than abolishing it, and preserve beneficial redox signaling (Sands et al. 2023; Villar et al. 2023).

#### Endogenous defense activators

The NRF2/KEAP1 pathway boosts the cell’s inducible antioxidant defenses, which is especially useful under chronic, low-grade oxidative stress. When NRF2 is released from KEAP1 and moves to the nucleus, it turns on a broad set of phase-II cytoprotective genes (Baird and Yamamoto 2020).

These include enzymes like heme oxygenase-1 (HO-1) and γ-glutamylcysteine synthetase (the rate-limiting step for glutathione synthesis), along with detoxifying and redox-buffering systems. The result is higher glutathione availability, improved removal of reactive species, and reinforced repair/clearance pathways—strengthening resilience without completely shutting down physiological redox signaling (Andrés et al. 2024b).

#### Metabolic and immunoredox reconditioning

Metabolic rewiring can shape redox tone and immunity by adjusting NADPH supply and the pathways that feed it. Enhancing or redirecting pentose phosphate pathway (PPP) flux increases NADPH generation, bolstering antioxidant systems (e.g., glutathione and thioredoxin cycles) and favoring resolution programs in immune cells. Conversely, throttling PPP output or diverting carbon can constrain NADPH and tilt cells toward pro-oxidant, inflammatory states (Britt et al. 2022; TeSlaa et al. 2023).

Likewise, tuning glutaminolysis—which supports anaplerosis and can fuel NADPH production via malic enzyme and IDH1/2—modulates mitochondrial substrate pressure, ROS formation, and effector functions (Ju et al. 2020). Together, interventions that nudge PPP activity, glutamine use, and related shuttles can recalibrate ROS buffering and tip immune responses toward resolution rather than sterile inflammation, without abolishing necessary microdomain redox signaling (Zhou et al. 2022).

Overall, clinical signals suggest efficacy depends on (1) choosing the source (NOX vs. mitochondria), (2) matching the compartment (membrane, mitochondrial matrix, peroxisome) and (3) respecting the disease phase (acute hyper-ROS vs. chronic redox dysfunction).

### Combined strategy with classic antioxidants

“Classic” antioxidants (e.g., vitamin C and E, NAC) show contextual benefits but heterogeneous outcomes when used indiscriminately. Evidence favors their use as adjuncts within defined pathophysiological frames:

- N-acetylcysteine (NAC)—Useful with glutathione deficit or conjugation toxicities and as rescue during ROS spikes; avoid chronic overuse that over-scavenges and flattens beneficial H_2_O_2_ signalling needed for proliferation/repair (Mason et al. 2020; Schwalfenberg 2021).
- Vitamin C—At pharmacologic i.v. doses can act locally pro-oxidant (Fenton-like) with potential anti-tumor cytotoxicity; at physiologic levels functions mainly as antioxidant and enzyme cofactor (Ngo et al. 2019).
- Vitamin E (tocopherols)—Stabilizes membranes against lipid peroxidation; requires recycling (e.g., ascorbate) to prevent tocopheroxyl radical buildup (Miazek et al. 2022).

Use generic antioxidants only after defining the redox phenotype (biomarkers + target compartments) and prioritize targeted (e.g., mitochondrial) or time-staged formulations (acute/subacute windows).

### Limitations and analytical challenges

Therapeutic control of ROS runs up against measurement limits that help explain many mixed outcomes. (1) A number of “ROS probes” have limited selectivity or yield side products; for example, dihydroethidium (DHE) can be oxidized to ethidium (E⁺) in addition to the diagnostic 2-hydroxyethidium (2-OH-E⁺), so raw fluorescence tends to overcall superoxide, and imaging can add phototoxicity and dose/time artifacts unless illumination and local chemistry are tightly controlled. (2) Plasma averages (e.g., 8-iso-PGF_2_α, protein carbonyls) rarely report on signaling microdomains (mitochondria–myofibril interfaces, caveolae, the nuclear envelope) where reversible cysteine oxidations encode transcriptional control; without compartment-targeted reporters, source/localization attribution is lost. (3) Reperfusion or immune bursts occur over milliseconds to seconds and are often missed ex vivo, necessitating time-stamped assays and, when feasible, low-phototoxicity in situ imaging/recording. (4) Standardization gaps, widely adopted SOPs are still lacking for preanalytics (time-to-spin, temperature, light/pH), metal-chelation stabilization and derivatization, plus reference materials and calibration curves reported alongside raw chromatograms/spectra; without that infrastructure, true biology is masked by ex vivo variability. These constraints can be mitigated by anchoring point-of-care readouts to validated lab assays, implementing specificity controls (e.g., SOD/catalase for O_2_·−/H_2_O_2_, chelators for Fenton dependence), and using compartment-targeted, ratiometric reporters with documented calibration (Król and Brzeźnicki 2023; Liu et al. 2020; Wu et al. 2019).

### Safety, contraindications and drug–drug interactions

Excessive, chronic suppression of ROS can be counterproductive: lowering oxidant tone too far may blunt adaptive angiogenesis, insulin signalling, osteogenesis and innate antimicrobial responses, potentially slowing tissue repair and increasing infection risk. Antioxidants may also antagonize pro-oxidant therapies—several chemotherapeutics and radiotherapy regimens rely on ROS generation for cytotoxicity—so indiscriminate use during active treatment can reduce efficacy; any supplementation should be timed and agreed with oncology teams (Ambrosone et al. 2020; Lennicke and Cochemé 2021).

Around exercise, high-dose peri-workout antioxidants attenuate hormetic training adaptations, including mitochondrial biogenesis and improvements in insulin sensitivity. Apply coadministration cautions: assess iron status (e.g., avoid or carefully justify i.v. vitamin C in iron overload due to Fenton chemistry), consider agents that shift redox balance (e.g., xanthine oxidase inhibitors altering ROS/RNS signalling) and be alert to myopathy risk when heavy antioxidant use coincides with high-dose statins. Overall, safety hinges on dosing to phenotype, respecting phase-of-disease timing and coordinating with therapies that intentionally leverage oxidative mechanisms (2022; Merry and Ristow 2016).

## Combination and sequential therapies based on ROS tuning

This section operationalizes ROS therapy as a time-staged sequence rather than a single intervention. We align tools to the source–sink–signaling triad: rapid source control (e.g., NOX/mitochondrial bursts) in the acute phase, set-point restoration and metabolic support in subacute recovery and maintenance/remodeling in chronic disease. Transitions are biomarker-guided (damage footprints, GSH/GSSG, protein sulfenylation, function) with planned titration and de-escalation to avoid over-scavenging and preserve physiological H_2_O_2_ signalling.

### Windows of opportunity: acute, subacute and chronic phases

Therapy should follow the time course of redox pathology, Table 2. In the acute window (0–24 h) the priority is to contain ROS spikes (e.g., ischemia–reperfusion, SIRS) by attenuating the source (NOX/mitochondrial bursts), buffering at the compartment of origin (mitochondria, ER, endothelium) and controlling free metals that license Fenton chemistry. In the subacute window (D2–14) the goal shifts to set-point restoration—re-establishing physiological H_2_O_2_ signalling, rebuilding antioxidant capacity (GSH/Trx/Prx) and supporting NADPH while avoiding over-scavenging. In the chronic window (≥ week 3) focus turns to maintenance/remodelling: preventing relapse, limiting fibrosis and endothelial dysfunction and leveraging lifestyle hormesis alongside gentle, compartment-targeted pharmacology. Transitions between windows are biomarker-guided (damage footprints down, signalling proxies back, redox and iron metrics normalized) and include planned titration/de-escalation to preserve beneficial H_2_O_2_ cues.

**Table 2:** Phase-specific ROS-guided therapy (acute, subacute, chronic): goals, dominant sources, key interventions and biomarkers

Phase (typical horizon)	Primary goals	Dominant sources/chemistry to address	Core tools and interventions	Biomarkers to enter/exit phase	Key cautions
Acute (0–24 h)	Contain bursts; limit ONOO^−^/·OH; stabilize endothelium & mitochondria	NOX2/NOX1 at membranes; mito RET (complex I) spikes; ER overflow; labile iron–driven Fenton	Rapid source control (NOX-directed strategies); mitochondria-targeted antioxidants; hemodynamic optimization; metal management (chelation where indicated); tight O_2_/Ca²⁺ control	Rising → falling damage footprints (F_2_-isoprostanes, protein carbonyls, nitrotyrosine); early Prx over-oxidation resolving; ↓ LIP (calcein-quench)	Avoid blanket antioxidants that erase signalling; watch hypotension/ischemia–reperfusion mismatch; chelation only with indication
Subacute (Day 2–14)	Restore redox set-point; re-establish physiological signalling; promote orderly repair/angiogenesis	Residual NOX4/ER H_2_O_2_ tone; mito matrix H_2_O_2_; post-injury RNS interplay	Moderate NRF2 activation; sustained SOD/catalase mimetics; support NADPH (PPP); NO/NOS re-coupling; begin targeted antioxidants (mitochondrial)	GSH/GSSG improving; Prx dimer/over-oxidation cycling normalized; protein sulfenylation (signalling proxy) re-appears; stable or falling F_2_-isoprostanes	Over-scavenging blunts plasticity and repair; coordinate with pro-oxidant oncologic/radiation regimens
Chronic (≥ Week 3)	Maintenance/remodelling; prevent fibrosis and endothelial dysfunction; resilience	Tonic NOX activity; ER/peroxisomal H_2_O_2_; iron handling drifting	Low-intensity NOX modulation; mitochondria-targeted maintenance; iron/ferroptosis hygiene (selenium/GPX4 support if needed); lifestyle hormesis (exercise, nutrition, sleep/circadian)	Stable composite panel (damage low; signalling present); functional gains (FMD, VO_2_); iron metrics normalized	Avoid long-term suppression of ROS tone; periodic de-escalation; monitor interactions (statins, xanthine-oxidase inhibitors, metals)

### Rational combinations: source–sink–signaling

Effective therapy pairs a source controller with an appropriate sink (detox/repair) while preserving signal (physiological H_2_O_2_ cues), Table 3. Combinations should be compartment-aware (mitochondria, ER, membrane microdomains), time-staged (acute → subacute → chronic) and biomarker-guided to avoid over-scavenging.

**Table 3:** Mechanism-guided combination therapies. (*Source* → Sink/Signal): Rationale, optimal window, tools, biomarker gates and cautions.

Combination (Source → Sink/Signal)	Rationale (what it fixes)	Best window	Typical tools (examples)	Biomarker gates (start/stop)	Key cautions
NOX-directed control → mito-targeted antioxidant	Quells membrane bursts and breaks feed-forward mito ROS while keeping basal H_2_O_2_ signalling	Acute → Subacute	NOX isoform modulation; mito-addressed scavengers	Fall in F₂-isoprostanes/carbonyls; recovery of Prx redox cycling	Don’t suppress phagocyte defense; avoid bulk antioxidant dosing
NRF2 activation (moderate) → lipid-peroxide “sink”	Replenishes enzymatic defenses and curbs peroxidation chains	Subacute	NRF2 modulators; SOD/catalase mimetics; RTA* support	Rising GSH/GSSG; ↓ lipid peroxides; preserved sulfenylation signal	Overactivation may blunt hormesis or aid tumors—use time-limited
Metabolic rewiring → localized antioxidant	Raises NADPH while protecting specific compartments, sustaining set-point without flattening signal	Subacute → Chronic	PPP/NADPH support; mito-targeted antioxidants; diet/exercise	↑ NADPH proxies; normalized Prx/thiol switches; functional gains	Avoid global scavenging; titrate with function (FMD/VO_2_)
Iron/LIP management → GPX4 support/RTAs	Lowers Fenton/ferroptosis drive; terminates lipid-radical chains	Acute (if LIP high) → Subacute	Chelation (context-specific), selenium/GPX4 support, RTAs	↓ LIP (calcein-quench); ↓ oxidized phospholipids	Chelators only with indication; watch anemia/infection risk
NOS re-coupling → selective NOX restraint	Restores NO signalling, cuts ONOO^−^ formation at endothelial/immune interfaces	Subacute	BH₄/arginine axis optimization; NOX1/2/4 targeting	↑ NO bioavailability (FMD); ↓ nitrotyrosine	Coordinate with BP meds; avoid excessive vasodilation
RSS augmentation → H_2_O_2_ signalling preservation	Persulfidation stabilizes thiol switches and recycles sulfenic acids without erasing H_2_O_2_ codes	Subacute → Chronic	Controlled-release H₂S/persulfide donors	Recovery of cysteine-switch readouts; stable Prx cycling	Dose to tissue set-point; monitor BP/mitochondrial effects
Source control (node-specific) → lifestyle hormesis	Fixes upstream chemistry and trains endogenous buffers for durable maintenance	Chronic	Node-specific drugs + structured exercise, nutrition, sleep	Stable composite panel; sustained functional metrics	High-dose antioxidants peri-exercise blunt training adaptations

### Disease archetypes and sequencing examples

Different disorders express distinct redox geometries—which sources dominate, where ROS act and how fast they fluctuate. Sequencing should therefore map to each archetype’s spike pattern (acute bursts vs. tonic tension), compartment (mitochondria, membrane NOX, ER/peroxisome) and iron/RNS context, with transitions gated by the composite biomarker panel and functional readouts. Table 4 summarizes pragmatic sequences, dosing and de-escalation should be titrated to phenotype, not symptoms alone.

**Table 4:** Disease archetypes and phase-sequenced ROS-guided care: windows (0–24 h, day 2–14, ≥ week 3), biomarker gates and key cautions

Archetype	Redox pattern (where/when)	0–24 h (Acute)	Day 2–14 (Subacute)	≥ Week 3 (Chronic)	Biomarker gates (advance/hold)	Special cautions
Ischemia–reperfusion (MI, stroke, major surgery)	Explosive bursts: NOX2/membrane + mito RET; labile iron surge	Rapid source control (NOX restraint), mito-targeted scavengers, hemodynamics, metal management	Set-point restoration: moderate NRF2, SOD/CAT mimetics, NADPH support, NOS re-coupling	Low-intensity NOX modulation; mito maintenance; lifestyle hormesis	↓ F₂-isoprostanes/carbonyls; Prx cycling recovers; LIP normalizes; FMD/VO_2_ trending up	Avoid bulk antioxidants; chelation only if indicated; coordinate anti-thrombotics/pressors
ARDS / critical illness	Mixed spikes + tonic oxidative tone; endothelial/immune interfaces	Source damping (NOX, ventilation strategy), iron hygiene; avoid hyperoxia	Controlled NRF2; antioxidant support without over-scavenging; nutrition/NADPH	Gradual de-escalation; rehab-oriented hormesis	Damage panel down; lactate/organ function improve; sulfenylation signal returns	Maintain host defense; watch DDIs with vasopressors/antimicrobials
Sepsis	Immune-driven ROS/RNS; endothelial leak; iron mobilization	Stabilize hemodynamics; cautious source control; metal handling; avoid flattening phagocyte ROS	Support redox enzymes; NOS re-coupling; mito support	Stepwise de-escalation; rehabilitation; microvascular function	↓ nitrotyrosine/oxidized lipids; FMD improves; GSH/GSSG rises	Do not suppress innate killing; antibiotic timing takes precedence
Fibrotic/metabolic disease (CKD, NAFLD/NASH, HFpEF)	Tonic NOX/ER/peroxisomal H_2_O_2_; low spikes; lipid peroxidation	Usually no “acute” stage; assess iron and basal tone	NRF2 (moderate), lipid-peroxide sinks (RTA), NOS re-coupling	Maintenance NOX restraint; mito-targeted low-dose; diet/exercise/sleep	Lipid peroxides down; stable sulfenylation; fibrosis/inflammation markers ease	Avoid long-term over-suppression; periodic drug holidays/retitration
Neurodegeneration (PD/AD/ALS)	Mitochondrial ROS + lipid peroxidation; ferroptosis propensity	If acute decompensation: gentle mito support; avoid deep ROS suppression	Mito-directed antioxidants; lipid-peroxide control; NADPH/mitophagy support	Long-horizon lifestyle hormesis; careful NOX maintenance	Oxidized phospholipids↓; Prx/thiol switches normalize; cognitive/motor metrics	Preserve synaptic plasticity; monitor sedation/DDIs
Atherosclerosis / endothelial dysfunction	NOX1/2/4, eNOS uncoupling; subendothelial lipid peroxidation	Address triggers (ischemia, ACS) as per I–R row	NOS re-coupling, NOX restraint, lipid peroxidation control	Maintenance with exercise, diet, sleep; mito-targeted support	↑ NO bioavailability (FMD); ↓ oxidized lipids; stable GSH/GSSG	BP interactions; statin-related myopathy with heavy antioxidant loads
Autoimmune/inflammatory (RA, IBD, SLE)	Immune NOX/RNS with episodic flares	During flare: targeted source control; protect endothelium/iron balance	Between flares: NRF2 (moderate), NADPH support; selective sinks	Maintenance lifestyle hormesis; cautious NOX modulation	Damage panel falls post-flare; functional scores improve	Coordinate with immunosuppressants; infection risk awareness
Oncology (supportive care; caution)	Context-dependent; many therapies use ROS	Do not blunt treatment-intended ROS; manage iron only if indicated	If allowed by oncology: time-boxed NRF2/antioxidants outside active therapy	Lifestyle and recovery; targeted support if permitted	Use oncology milestones; monitor nitrotyrosine/oxidized lipids	Always schedule with oncology; avoid peri-chemo/radiation over-scavenging

### Biomarkers and titration

Titration should follow a composite, compartment-aware panel that pairs species/flux readouts with cumulative damage footprints, buffer status, iron/ferroptosis metrics and functional endpoints. Use species-level signals (e.g., DHE-HPLC for O_2_·− via 2-OH-E⁺, compartment-targeted HyPer/roGFP-Orp for H_2_O_2_, peroxiredoxin redox state as a fast sentinel) to attribute source and location, use footprints (F₂-isoprostanes, protein carbonyls/nitrotyrosine, 8-oxoG) to gauge exposure, add buffer indices (GSH/GSSG, TAC) and iron axis (labile iron pool, oxidized phospholipids, GPX4 context) to estimate ·OH/ferroptosis propensity. Transitions are biomarker-gated: move from acute → subacute when damage markers fall and signalling proxies (sulfenylation/Prx cycling) re-emerge, progress to chronic when redox buffers and function normalize. De-escalate if over-suppression appears (flattened Prx dynamics, loss of signalling proxies) or if iron-licensed peroxidation rises despite low bulk ROS. Always report compartment and time for each assay and anchor dosing to the panel—not symptoms alone, Table 5.

**Table 5:** Biomarker panel to gate ROS-guided therapy: domains, preferred assays, clinical use, advance/hold criteria and notes

Biomarker domain	Exemplary assays (preferred methods)	What it tells you	When it’s most useful	Gateposts to advance / hold	Notes & cautions
Species / flux (source attribution)	O_2_·− by DHE→2-OH-E⁺ (HPLC); compartment-targeted HyPer/roGFP-Orp (matrix/IMS/ER/nucleus); Prx redox state (non-reducing gels)	Real-time ROS flux and compartment; Prx cycling = buffer load	All phases; mechanistic sub-studies	Advance when Prx over-oxidation resolves and targeted H_2_O_2_ pulses return; Hold if persistent high flux from NOX/mito sites	Avoid raw DHE fluorescence; correct HyPer for pH; always state compartment/time.
Damage footprints	F₂-isoprostanes (LC-MS/MS), oxidized phospholipids; protein carbonyls, 3-nitrotyrosine; 8-oxoG / DNA breaks	Cumulative injury burden and pathway (lipid/protein/DNA; RNS)	Acute→Subacute transitions; safety monitoring	Advance when footprints trend down consistently; Hold/step back if they rise despite flux control	Prefer LC-MS/MS over colorimetry; validate nitrotyrosine specificity.
Redox buffer status	GSH/GSSG, total antioxidant capacity; NADPH/NADP⁺ proxies; Trx/Prx cycling	System readiness to decode H_2_O_2_ signals without spillover	Subacute recovery	Advance when GSH/GSSG normalizes and Prx cycling resumes; Hold if buffers remain depleted	Interpret with species data to avoid mistaking high TAC for health.
Iron / ferroptosis axis	Labile iron pool (calcein-quench ± chelator reversibility); ferritin/HO-1/NCOA4 status; GPX4/selenium context; lipid peroxides	Risk of Fenton ·OH and ferroptosis	Acute (iron surge) and fibrotic/metabolic settings	Advance when LIP and oxidized lipids fall; Hold/chelate if LIP high or oxPLs rise	Use chelation only with indication; track anemia/infection risk.
Signalling proxies	Protein sulfenylation (“switch” assays), oxidized-PTP signatures, pathway phospho-readouts	Return of physiological H_2_O_2_ signalling	Gate subacute→chronic	Advance when signalling proxies re-appear alongside low damage	Pair with species/compartment data to avoid misattribution.
Functional endpoints	Endothelial FMD, VO_2_ peak/submax, HRV, organ scores	Systems-level recovery and safety	Late subacute and chronic	Advance/de-escalate when function improves stably	Use alongside biochemical panel; function alone is insufficient.
Reporting discipline	SOPs for sampling, metal chelation, derivatization; timestamping	Data quality and comparability	Always	—	Always report species, compartment, timing and controls.

### Implementation checklist

Redox therapy should follow a short, repeatable cycle: phenotype → target → deliver → monitor → (de-)escalate. Use a composite, compartment-aware panel and make every change biomarker-guided rather than symptom-led, Table 6.

**Table 6:** Stepwise implementation for ROS-guided therapy: phenotype → target → deliver → monitor → (De-)escalate—steps, tools, decision rules and pitfalls

Step	What to do	Tools / assays (examples)	Decision rule (advance/hold)	Common pitfalls
1. Phenotype	Map species, source, compartment, timing	DHE→2-OH-E⁺ (HPLC) for O_2_·−; compartment-targeted HyPer/roGFP-Orp for H_2_O_2_; Prx redox state	Proceed when a dominant source and locale are identified	Using raw probe fluorescence; ignoring compartment/time
2. Baseline burden	Quantify damage and iron context	F₂-isoprostanes/oxPLs, carbonyls, nitrotyrosine; LIP (calcein-quench ± chelator), ferritin/HO-1/NCOA4	Treat if damage > baseline or LIP elevated	Treating numbers without linking to species/source
3. Choose node	Pick source-first intervention; plan the sink; preserve signal	NOX/NOS modulation; mito control (RET/Ca²⁺); ER redox tuning; iron/ferroptosis levers; RSS support	Start with lowest effective node; add sink if damage persists	Blanket antioxidants that flatten H_2_O_2_ signalling
4. Deliver by phase	Match therapy to acute / subacute / chronic window	See 12.1–12.3 modules	Advance window when damage falls and signalling proxies return	Staying “acute” too long; no planned de-escalation
5. Titrate dose/route	Anchor to panel deltas, not weight alone	GSH/GSSG, Prx cycling, targeted reporters; FMD/VO_2_	Up-titrate if flux/damage remain high; down-titrate if signalling flattens	Chasing symptoms without biomarker confirmation
6. Check DDIs/safety	Reconcile with pro-oxidant therapies and exercise	Treatment calendar; oncology/radiation timing; training blocks	Pause or time-box antioxidants if they oppose intended ROS	Peri-exercise megadoses; peri-chemo scavenging
7. Reassess iron axis	Prevent Fenton/ferroptosis ignition	LIP, oxidized phospholipids, GPX4/selenium status	Add chelation/RTAs/selenium only if LIP/oxPLs high	Indiscriminate chelation → anemia/infection risk
8. Document & report	Ensure SOPs + timestamps for comparability	Sampling matrix, chelators, derivatization, acquisition timing	Report species, compartment, time for every readout	Pooled/averaged signals with no attribution

In summary, Tables 2, 3, 4, 5 and 6 build a practical, end-to-end playbook for “tuning” ROS rather than bluntly suppressing it. Table 1 lays out a phase-specific scaffold—acute, subacute, chronic—that aligns therapeutic goals, dominant sources and biomarker-gated transitions across windows of time. Table 2 then pairs “source” control with the right “sink/repair” while preserving physiological signalling (source→sink/signal), emphasizing compartment awareness and time-staged use of tools to avoid over-scavenging.

Table 3 translates this into disease-level sequences by archetype (e.g., ischemia–reperfusion, ARDS, fibrotic/metabolic disease), mapping interventions to each pattern’s burst/tonic geometry and specifying advance/hold gates.

Table 4 specifies the composite biomarker panel—species/flux, damage footprints, buffer status, iron/ferroptosis axis, signalling proxies and functional endpoints—and codifies how those readouts trigger escalation, de-escalation and phase transitions.

Finally, Table 5 operationalizes everything into a short, repeatable cycle—phenotype → target → deliver → monitor → (de-)escalate—anchoring dosing and combinations to the panel rather than symptoms alone.

## Knowledge gaps and future directions in redox biology

We still lack species-resolved, compartment-aware and calibrated measurements in intact tissues and humans. Most clinical and many preclinical readouts are footprints (e.g., lipid peroxidation) that blur source, timing and location. The field needs validated, in vivo–compatible flux sensors (O_2_·−, H_2_O_2_, NO/ONOO^−^) targeted to matrix/IMS, ER lumen, peroxisomes, plasma-membrane microdomains and nucleus, each with built-in specificity controls and reporting standards (matrix, oxygen tension, ΔΨm, pH) to disambiguate signalling pulses from damage plateaus. A practical step is to formalize panelized workflows that combine species reporters + footprints + iron metrics and to require calibration and reversibility tests (e.g., SOD/catalase, chelators) in publications and trials (Byrne et al. 2015; Herb et al. 2021; Hoehne et al. 2022).

## Spatial biology and contact-site mapping

ROS biology is orchestrated at organelle contact sites (MAMs, peroxisome–mitochondria, mitochondria–lysosome) and membrane microdomains, yet we lack tools to image, perturb and quantify multi-reactive chemistry there at cellular resolution in vivo. Future work should pair super-resolution and functional imaging with organelle-addressed reporters, localized perturbations (isoform-specific NOX/NOS, ER oxidoreductases, RET modulators) and computational models that capture vectorial flow rather than free diffusion. Building reference atlases of contact-site ROS/RNS/RSS dynamics across tissues, ages and disease stages is a tractable community goal (Kwak et al. 2020; Wong et al. 2018).

## Iron, ferroptosis and lipid redox homeodynamics

We can measure the labile iron pool (LIP) in cells, but spatiotemporal LIP mapping in vivo—including ferritinophagy pulses, heme turnover and mitochondria–lysosome exchange—remains rudimentary. We need quantitative links between local LIP, PUFA-PL composition (ACSL4/LPCAT3) and anti-ferroptotic circuits (GPX4, FSP1/DHODH), to predict where/when H_2_O_2_ becomes Fenton-competent and when ferroptosis ignites. Validated lipid-peroxide signatures paired with iron handling markers should be standardized for clinical studies (Bersuker et al. 2019).

## Crossing the streams: ROS–RNS–RSS reciprocity

Chemical interconversion and crosstalk mean single-species conclusions are fragile. Future platforms must co-measure O_2_·−/H_2_O_2_, NO/ONOO^−^ and RSS/persulfides with kinetic discrimination and compartment targeting, then causally separate nodes using genetic/pharmacologic tools (NOX/NOS isoforms, trans-sulfuration enzymes) under pre-registered control sets. This is essential to avoid conflating correlated but distinct chemistries in both discovery biology and trials (Zuckerman et al. 2017).

## From models to humans: external validity

Rodent phenotypes often reflect housing, diet and circadian conditions that reshape redox tone. We need human-proximal systems (patient-derived organoids, microphysiological chips, single-cell redox omics) validated against clinical panels (species → footprint → iron) and functional outcomes. Longitudinal human studies should deliver reference ranges by matrix (plasma, CSF, urine, breath), sex, age and disease stage and explicitly anchor POC trends to lab-grade assays during adoption (Nair et al. 2025).

## Trial design: mechanism-anchored endpoints

Too many studies report “ROS lowering” without benefit. Trials should co-primary mechanistic change (e.g., Prx cycling, 2-OH-E⁺ by DHE-HPLC, LIP de-quench) and clinical improvement, with biomarker gates for entry and adaptive dosing to respect “where, when, how much.” Pre-specified normalization (per protein/cell, oxygenation, ΔΨm for mito-probes) and orthogonal controls (SOD/catalase, chelators, genetics) should be mandatory (Katerji et al. 2019).

## Therapeutic specificity and safety

Node-specific strategies outperform blanket scavenging, but on-target physiology can be collateral damage if dosing is not time-stamped and compartment-aware. Open questions include the long-term safety of NOX/NRF2 modulation, NOS re-coupling in multimorbidity, RET tempering without blunting mitohormesis, ER redox tuning under chronic secretory stress and RSS donors with controllable release and organelle targeting. Each requires biomarker-guided windows that preserve physiological H_2_O_2_ cues while suppressing ONOO^−^/Fenton hotspots (Cipriano et al. 2023a; Dinkova-Kostova and Copple 2023).

## Data and computation

We need integrative analytics that fuse species-level flux, footprints and iron propensity with imaging, proteo-lipidomics and function. Community datasets and open pipelines (with raw chromatograms/spectra) would accelerate mechanistic inference and model-based dose selection. Ultimately, decision support should move from “antioxidants” to node/compartment prescriptions personalized by redox phenotypes (Wilkinson et al. 2016).

##  A prioritized roadmap

A practical roadmap to advance redox medicine starts with standards and panels: finalize community reporting standards and define minimal effective panels—linking species → footprint → iron propensity—for each clinical indication so that measurements are comparable across labs and directly actionable.

Next, invest in contact-site biology by creating tools and reference atlases that resolve microdomain redox at organelle interfaces (MAMs, peroxisome–mitochondria, mitochondria–lysosome) and plasma-membrane nanodomains, because that is where signalling turns into damage (Xia et al. 2024).

Tackle the iron–lipid axis with in vivo mapping of the labile iron pool (LIP) and validated ferroptosis risk assays that couple iron handling (ferritin/ferritinophagy, HO-1) to lipid-peroxide signatures, predicting when H_2_O_2_ becomes Fenton-competent and chains ignite. Bridge models to patients by aligning human-proximal systems—organoids, microphysiological chips and point-of-care (POC) modalities—with lab-grade validation (chromatography/spectrometry, calibrated reporters) to ensure external validity. Fifth, redesign trials as mechanism-anchored: use biomarker gates for enrollment and co-primary endpoints that pair mechanistic change (e.g., Prx cycling, 2-OH-E⁺, LIP shifts) with clinical benefit, avoiding the trap of “ROS lowering without outcome” (Drakulic et al. 2022).

Finally, define therapy windows—explicit dose–time–compartment rules—for node-specific interventions across NOX/NOS, mitochondrial (RET/ΔΨm/Ca²⁺), ER (UPR^ER/Ero1–PDI), iron/ferroptosis (LIP/GPX4/FSP1/DHODH) and RSS (H₂S/persulfides), so treatments preserve physiological H_2_O_2_ signalling while preventing convergence onto ONOO^−^, Fenton ·OH, or runaway lipid peroxidation. Together, these steps operationalize our organizing principle—where, when and how much—and carry it from bench to bedside (de Almeida et al. 2022).

## Conclusions

ROS are neither uniformly harmful nor uniformly beneficial; their biological impact is decided by where they arise (source and subcellular compartment), when they occur (brief pulses versus sustained plateaus) and how much oxidant is delivered. Across mitochondria, NOX enzymes, ER and peroxisomes, physiological H_2_O_2_ microgradients encode signalling via reversible cysteine chemistry, whereas spillover—especially in iron-rich niches—tips reactions toward peroxynitrite, Fenton-derived ·OH, lipid peroxidation and regulated cell death. This where/when/how-much rule reconciles the dual nature of ROS in health and disease and explains why indiscriminate “antioxidant loading” so often disappoints.

Methodologically, progress hinges on species-resolved, compartment-aware analytics coupled to standardized reporting. Panels that pair flux readouts (e.g., DHE→2-OH-E⁺ for O_2_·−, compartment-targeted HyPer/roGFP-Orp for H_2_O_2_, peroxiredoxin redox state) with damage footprints (F₂-isoprostanes, carbonyls/nitrotyrosine, 8-oxoG) and iron/ferroptosis metrics (labile iron pool, oxidized phospholipids, GPX4 context) provide the attribution needed to separate signalling from injury, compare studies and titrate therapy.

Therapeutically, the evidence favors node-specific, compartment-targeted interventions—NOX/NOS modulation, mitochondrial quality control and RET tempering, ER redox tuning, calibrated sulfur-axis support and iron/ferroptosis management—implemented as time-staged sequences (acute → subacute → chronic) and guided by biomarker gates rather than symptoms alone. Such strategies preserve beneficial H_2_O_2_ signalling while preventing convergence onto ONOO^−^ formation, hydroxyl radical chemistry and runaway lipid peroxidation. The practical message is simple: treat the map, not the mean—identify which species, where and when, intervene at the responsible node and compartment and de-escalate once signalling recovers and damage abates.

Finally, we see clear priorities for the field: consensus SOPs and reference materials, routine compartment/time stamps in datasets, better in-vivo iron/ferroptosis assessment and mechanism-anchored, biomarker-guided clinical trials. Adopting these standards will convert redox biology from descriptive correlations into precision modulation with direct translational impact.
